# Tumor Topography Remodels TME Signalling via Compartment-Specific Host–Microbiome–Tumor Crosstalk in Hepatoblastoma

**DOI:** 10.3390/cancers18172854

**Published:** 2026-09-03

**Authors:** Beate Obermüller, Sabine Obermüller, Karin Wagner, Maximilian Nepel, Holger Till, Georg Singer, Bettina Leber

**Affiliations:** 1 Department of Paediatric and Adolescent Surgery, Medical University of Graz, 8010 Graz, Austria; 2 HEALTH—Institute for Biomedical Research and Technologies, Joanneum Research Forschungsgesellschaft mbH, 8010 Graz, Austria; 3 Center for Medical Research, Medical University of Graz, 8010 Graz, Austria; 4 Department of Gastroenterology and Hepatology, Medical University of Graz, 8010 Graz, Austria; 5 Division of General, Visceral and Transplant Surgery, Department of Surgery, Medical University of Graz, 8010 Graz, Austria

**Keywords:** hepatoblastoma, TME, bacterial and archaeal microbiome, transcriptomics

## Abstract

**Background & Aims**: Signaling within the tumor microenvironment (TME) emerges from dynamic crosstalk among cancer cells, host tissues, and the microbiome. Orthotopic and ectopic xenograft models are widely used in preclinical liver cancer research, yet it remains unclear to what extent tumor location shapes systemic host responses and gut–liver communication. We therefore investigated how anatomical context influences host transcriptomes and spatially resolved intestinal microbial ecosystems in hepatoblastoma (HB). **Methods**: We integrated bacterial/archaeal ecology with host and xenograft transcriptomes in orthotopic versus ectopic hepatoblastoma xenografts in male athymic CAnN.Cg-Foxn1nu/Crl mice. Single-sample GSEA, sparsity-aware networks, and DIABLO multi-block integration mapped pathway-level coordination across intestinal segments, stool, secondary lymphoid organs, livers, and tumors. **Results**: Tumor localization was associated with compartment-specific differences in host signaling, with the mesenteric lymph node and liver showing prominent shifts in TNFA/NF-κB, hypoxia, unfolded protein response, xenobiotic metabolism, mTORC1, KRAS, epithelial–mesenchymal transition, and MYC/E2F cell-cycle axes. Archaea exhibited pronounced, niche-specific signatures that showed positive correlations with proliferative and inflammatory Hallmarks and negative correlations with interferon pathways. Multi-omics integration (r ≥ 0.85) indicated coordinated variation linking specific microbial families to host and tumor programs and positioned the xenograft block as a hub connecting EMT, mTORC1, and angiogenesis to defined archaeal and bacterial taxa. **Conclusions**: Tumor topography remodels TME signaling through compartment-dependent host–microbiome–tumor crosstalk, with archaeal domains emerging as salient correlates of proliferative and stress-response networks. These findings provide a systems framework and testable hypotheses for therapeutic modulation of TME signaling via interkingdom interactions.

## Simple Summary

Tumors develop within a complex microenvironment shaped by signals from cancer cells, host tissues, and the microbiome. We combined bacterial and archaeal profiling with host and tumor (xenograft) transcriptomes in orthotopic versus ectopic hepatoblastoma models to examine how tumor location relates to signaling programs in the tumor microenvironment (TME). Using pathway-level analyses and multi-omics integration, we observed compartment-specific differences in key signaling axes, including TNFA/NF-κB, hypoxia, the unfolded protein response, xenobiotic metabolism, mTORC1, KRAS, epithelial–mesenchymal transition, and MYC/E2F cell-cycle programs. Archaea showed pronounced, niche-specific signatures that correlated positively with proliferative and inflammatory pathways and negatively with interferon responses. These results provide a compartment-resolved map of host–microbiome–tumor crosstalk at the signaling level and generate hypotheses for modulating TME signaling through interkingdom interactions.

## 1. Introduction

Hepatoblastoma (HB) is the most common primary malignant liver tumor of childhood, accounting for approximately 70–80% of pediatric hepatic malignancies yet representing less than 1% of all childhood cancers. Despite its rarity, HB constitutes a major cause of cancer-related morbidity and mortality in early childhood [1,2,3]. The disease predominantly affects infants and toddlers, with most cases diagnosed before the age of five years and a median age at diagnosis of approximately 18 months [4]. The increasing incidence observed over recent decades has been partially attributed to improved survival of very preterm and low-birth-weight infants—a critical clinical cohort characterized by disrupted perinatal exposures, early-life gut dysbiosis, and immature immunometabolic development [5]. Despite advances in multimodal therapy, HB remains a relevant pediatric malignancy and its systemic biological and environmental contributors remain incompletely understood [6,7,8].

Over the past three decades, risk-adapted treatment strategies combining cisplatin-based chemotherapy, surgical resection, and, in selected cases, liver transplantation have substantially improved clinical outcomes. Five-year overall survival rates now exceed 80–90% in patients with localized, standard-risk disease [3]. However, outcomes remain worse in high-risk subgroups, including patients with metastatic disease, Pre-Treatment Extent of tumor (PRETEXT) IV tumors, vascular invasion, low alpha-fetoprotein expression, or unfavourable histological subtypes. Therapy-associated toxicities, particularly cisplatin-induced ototoxicity and long-term organ dysfunction, highlight the need for a deeper biological understanding of tumor behavior beyond clinical risk stratification alone. These limitations underscore that progress will likely depend on shifting from a purely tumor-centric perspective toward host–tumor interactions [3,8,9,10,11].

At the molecular level, HB is characterized by a low somatic mutation burden compared to most adult malignancies [12]. Despite this apparent genetic simplicity, the disease exhibits pronounced biological heterogeneity, suggesting that tumor behavior is not primarily driven by mutational complexity but also by dysregulated developmental programs [12]. Recent multi-omics and single-cell analyses support the concept that HB represents a developmental malignancy arising from aberrant persistence of fetal hepatoblast-like transcriptional states [13,14]. A relatively small number of genetic alterations can induce profound transcriptional, epigenetic, and metabolic reprogramming reminiscent of early liver organogenesis [14]. Because early hepatic organogenesis is linked to systemic metabolic maturation and mucosal homeostasis, these oncogenic circuits are likely embedded within a broader multi-compartment network of the developing host [13,14,15].

The canonical Wnt/β-catenin signaling pathway represents the central molecular hallmark of HB. Activating mutations in *CTNNB1*, most frequently affecting exon 3, as well as less frequent alterations in *AXIN1* or *APC*, result in stabilization and nuclear accumulation of β-catenin [16,17,18,19]. This leads to constitutive activation of TCF/LEF-dependent transcriptional programs that promote proliferation, inhibit differentiation, and maintain fetal cellular identity [16,20]. However, Wnt/β-catenin activation alone is insufficient to fully explain HB heterogeneity and aggressiveness [14,15]. Cooperative activation of additional developmental pathways, particularly Hippo signaling through nuclear YAP/TAZ, has been shown to synergize with β-catenin in tumor initiation and progression [21,22]. Experimental models have further implicated combined activation of these pathways in HB-like tumor formation [23].

Beyond these core developmental drivers, additional signaling networks including PI3K/AKT/mTOR, MYC, NRF2-mediated oxidative stress responses, and insulin-like growth factor signaling contribute to tumor growth, metabolic adaptation, and therapeutic resistance. These pathways are tightly interconnected and establish a transcriptional and metabolic state that supports rapid proliferation while maintaining resistance to oxidative and metabolic stress. HB biology is also shaped by epigenetic remodeling, including DNA methylation changes, chromatin accessibility alterations, and non-coding RNA-mediated regulation. Together, these processes stabilize fetal-like transcriptional programs despite the low mutational burden of the disease [14,18,20].

Concomitantly, HB development is influenced by its surrounding tumor microenvironment (TME) [23]. The TME comprises immune cells, endothelial cells, stromal fibroblasts, extracellular matrix components, and soluble signaling molecules [24,25]. These components engage in continuous bidirectional communication with tumor cells, influencing tumor progression, immune evasion, angiogenesis, and therapeutic response [25]. In this context, the role of the innate immune apparatus and myeloid cell lineages within the liver stroma remains underappreciated [26]. These interactions suggest that HB should be viewed not as a tumor cell-autonomous disease, but as a multicellular system shaped by malignant and non-malignant compartments [20,23].

The liver occupies a unique anatomical and immunological position due to its direct connection to the gastrointestinal tract via the portal circulation. This gut–liver axis exposes hepatic tissue to microbial products, metabolites, bile acids, and dietary-derived molecules. In physiological conditions, this system contributes to immune tolerance and metabolic homeostasis. However, disruption of intestinal microbial composition or barrier function can alter hepatic immune activation and inflammatory signaling. In adult liver diseases and hepatocellular carcinoma, dysregulation of the gut–liver axis influences disease progression, immune responses, and metabolic remodeling. In contrast, its role in pediatric liver tumors, including HB, remains largely unexplored [27,28,29,30].

The intestinal microbiome is spatially and functionally structured rather than a homogeneous entity. Microbial communities in the ileum and colon differ with respect to oxygen availability, nutrient gradients, bile acid exposure, and host immune interactions [31,32,33]. These compartments therefore generate distinct metabolic and immunological outputs that may differentially influence hepatic physiology through the portal circulation [34,35]. Reliance on stool samples alone may therefore obscure region-specific mucosal microbial signals that are most relevant for liver exposure [32].

Moreover, the intestinal microbiome extends beyond bacterial populations to include archaeal communities. Despite their evolutionary antiquity and distinct metabolic wiring, archaea have been largely omitted from oncological frameworks due to standard sequencing constraints. Yet, archaeal families may contribute to microbial metabolic networks, particularly in hydrogen consumption, interspecies fermentation, and methanogenesis [36,37]. Their potential contribution to host physiology, systemic immune tone, and cancer-related processes remains largely unexplored [36].

Recent evidence from multiple cancer entities suggests that microbial-derived metabolites, including short-chain fatty acids, secondary bile acids, and gasotransmitters, can modulate key oncogenic pathways such as Wnt/β-catenin, Hippo/YAP, PI3K/AKT, and inflammatory signaling cascades [17,34,38]. These observations support the emerging concept that microbial ecosystems are not passive bystanders but active regulators of host signaling networks [31]. Given the anatomical proximity and functional interdependence of the intestine and liver, region-specific microbial and archaeal communities may contribute to shaping the hepatic TME and influencing tumor biology [17,31,34,35].

Despite these converging lines of evidence, a comprehensive systems-level integration of tumor-intrinsic signaling, tumor-microenvironmental interactions, host organ responses, and region-specific gut microbiome composition has not yet been systematically performed in HB. In particular, the potential cross-talk between embryonic developmental signaling pathways, systemic host adaptations, and spatially distinct intestinal microbial ecosystems remains completely unmapped.

Against this background, we investigated HB as an integrated, multi-compartment biological system using orthotopic and ectopic human HuH6 xenograft mouse models. By integrating human-specific molecular profiling of tumor tissue with transcriptional analyses of five host organs (liver, spleen, mesenteric lymph nodes, lung, and kidney) and characterization of region-specific intestinal microbiomes—capturing both bacterial and archaeal communities across the ileum, colon, and feces—we aimed to capture the global architecture of tumor–host–microbiome interactions.

To resolve how these distinct physiological layers interact under host adaptive immune constraints, our study utilized male athymic CAnN.Cg-Foxn1nu/Crl mice, providing a setting to isolate innate immune and myeloid regulatory dynamics without T-cell interference [39]. High-dimensional data blocks were integrated using the mixOmics DIABLO framework to construct cross-omics interactomes.

We hypothesized that HB is shaped not only by tumor-intrinsic genetic events, but also by a dynamic network of developmental signaling, organ-specific host responses, and gut-derived microbial and archaeal influences that together shape tumor biology within a coordinated multi-organ ecosystem.

## 2. Materials and Methods

### 2.1. Animals

Following approvals of the Austrian veterinary board (2024-0.307.647: 30 April 2024 and 2025-0.281.987: 18 April 2025) and in accordance with the ARRIVE guidelines (checklist please see Supplementary File S1), 18 male athymic CAnN.Cg-Foxn1nu/Crl mice were obtained at an age of 6 weeks from Charles River Laboratories (Wilmington, MA, USA). Mice were kept single-housed in specific pathogen-free (SPF) conditions in individually ventilated cages (IVC) with a 12 h light–dark cycle and ad libitum irradiated standard diets (V1534-703, ssniff Spezialdiaeten GmbH, Soest, Germany) and autoclaved water at all times. After an adaptation phase of 2 weeks animals were allocated to 3 groups with 6 animals each, aiming for equal body weight distribution. During the whole experiment animals were checked daily, and the body weight, the food weight and a general health condition score were measured twice a week.

### 2.2. Cell Culture

Human HB cells (HuH6 clone 5) were obtained from the Japanese Collection of Research Bioresources Cell Bank (JCRB0401 HuH6 Clone 5, Osaka, Japan). Cells were thawed to room temperature and cultured in 75cc flasks with ventilated caps in Dulbecco’s modified Eagle’s medium (DMEM, Gibco, Waltham, MA, USA) with 4.5 g/L high glucose and 4 mM glutamine supplemented with 10% fetal bovine serum (FBS, Sigma Aldrich Handels GmbH, Vienna, Austria). 1% PenStrep (Carl Roth GmbH + Co. KG, Karlsruhe, Germany) was added to prevent bacterial overgrowths. HuH6 cells had a doubling time of approximately 2 days. Cells were passaged and split when reaching 85–90% confluence. Cells were harvested using a cell scraper (Ref 353085, BD Falcon, Bedford, MA, USA). For inoculation, a suspension of HuH6 cells in media without PenStrep and FBS with a concentration of 1.5 million cells per 0.1 mL was prepared. Cells were negatively tested against Mycoplasmas and the identity of the cells was confirmed via STR analysis right before inoculation.

### 2.3. Tumor Cell Inoculation

The ectopic tumor group (ectopic, *n* = 6) received 2 depots containing 1.5 million HuH6 cells in 0.1 mL of the medium without FBS or PenStrep each subcutaneously next to the knee. The orthotopic tumor group (orthotopic, *n* = 6) received 1 depot containing 1.5 million HuH6 cells in 0.02 mL of the medium without FBS or PenStrep directly into one liver lobe. In the control group (control, *n* = 6), 3 animals received 0.1 mL of the medium without FBS or PenStrep per depot subcutaneously in the corresponding area and 3 mice received 0.02 mL of the medium without FBS or PenStrep in one liver lobe.

### 2.4. Sampling

Tumors were allowed to grow for up to 15 weeks depending on the size of the tumor and the general condition of each mouse. Three hours before tissue collection, every animal received 400 µL of isotonic saline subcutaneously and fresh stool samples were taken directly from the anus. Anesthesia was performed in an induction chamber (Rothacher Medical GmbH, Heitenried, Switzerland) using Isoflurane (Forane^®^, Baxter Healthcare GmbH, Vienna, Austria) with approximately 4 vol% isoflurane and 1.5 L of oxygen per minute. After checking the depth of anesthesia via reflexes, as much blood as possible was taken via cardiac puncture and transferred to standard serum vials. Afterwards, cervical dislocation was performed and the liver, lung, mesenteric lymph node, kidney, spleen and the tumor itself were removed, weighted, aliquoted, snap frozen in liquid nitrogen and stored at −80 °C for further investigation. Additionally, stool samples of the ileum and the colon were taken from the corresponding sections of the intestine and snap frozen in liquid nitrogen and stored at −80 °C as well. Blood was allowed to clot for 30 min and then centrifuged for 10 min at 11,000× *g*. Supernatant serum was aliquoted and frozen at −80 °C for further investigations.

### 2.5. RNA Library Preparation and Sequencing

For each tissue, a defined amount (liver, spleen, tumor: 5 mg; kidney: 10 mg; lung: 20 mg; 1 lymph node) was homogenized in a MagNA Lyser Green Bead tube (Roche Holding, Basel, Switzerland) and the miRNeasy Kit (Quiagen, Venlo, The Netherlands) was used for RNA extraction according to the manufacturer’s instructions. The elution volume was also adjusted for each tissue: lymph node and lung: 15 µL; all others: 30 µL. The RNA concentration of each sample was evaluated using 1 µL on a NanoDrop (Thermo Fisher Scientfic, Wilmington, NC, USA).

RNA integrity was assessed using the RNA 6000 Nano LabChip on an Agilent 2100 Bioanalyzer system (Agilent Technologies, Foster City, CA, USA). RNA integrity numbers (RIN) ranged from 2.1 to 9.4 across all samples.

Libraries were prepared from 400 ng total RNA using the QuantSeq 3′ mRNA-Seq V2 Library Prep Kit FWD with UDI 12nt indexes (Lexogen GmbH, Vienna, Austria) according to the manufacturer’s protocol. Since the workflow targets polyadenylated RNA, ribosomal RNA depletion was not performed. Bead purification steps were performed in a single batch using a VIAFLO96 liquid handling system (INTEGRA Biosciences AG, Zizers, Switzerland). The required library amplification cycles were determined using the PCR Add-on and Reamplification Kit V2 (Lexogen GmbH), resulting in 10 PCR cycles for most libraries and 14 cycles for five samples.

Final libraries were quantified using a Quantus Fluorometer with the QuantiFluor ONE dsDNA System (Promega, Madison, WI, USA), and fragment size distribution was assessed using an Agilent 2100 Bioanalyzer with the DNA High Sensitivity LabChip. Libraries showed a mean fragment size of 362bp. Samples were pooled using 7 ng per library. Due to limited tissue availability, low RNA quality (RIN < 4.5), and amplification failure, sample sizes varied across analyses. For microbiome and archaeome profiling, the sample sizes were orthotopic (*n* = 5), ectopic (*n* = 6), and control (*n* = 6). For GSEA, ssGSEA, and DIABLO analyses, sample sizes for kidney, lung, liver, and spleen tissues were orthotopic (*n* = 5), ectopic (*n* = 6), and control (*n* = 6); for lymph nodes, they were orthotopic (*n* = 3), ectopic (*n* = 5), and control (*n* = 5); and for tumor tissues, which were absent in control animals by definition, they were orthotopic (*n* = 4) and ectopic (*n* = 6).

The pooled library was additionally purified using 0.9× SPRIselect beads (Beckman Coulter, Brea, CA, USA) and sequenced at the Vienna BioCenter Core Facilities GmbH (Vienna, Austria) on one lane of an Illumina NovaSeq X 10B platform (Illumina, San Diego, CA, USA) using paired-end 150 bp sequencing. FASTQ read 1 files were processed using Kangooroo beta, including adapter trimming, UMI collapsing, read quality assessment, and gene counting (DAP v1.7.9; cutadapt v1.18; umicollapse v1.1.0; RSeQC v5.0.1; featureCounts v1.6.4).

Sequencing generated 10.11–18.79 million raw reads per sample (LK-ctrl-085: 8.34 million reads). Additional quality control, correlation analysis, clustering, and differential expression analysis were performed in Galaxy (25.0.5.dev0) using ggplot2-based visualization, correlation analysis, heatmap2, and DESeq2 with default normalization settings and parametric fitting. RNA integrity was generally preserved across tissues, with median RIN values ranging from 6.0 to 7.9. Only five of 93 analyzed samples had RIN values below 4.0, limited to lymph node and tumor samples; no samples had RIN values between 4.0 and 5.0 (Supplementary Table S5).

### 2.6. Microbiome and Archaeome

All stool samples were sent on dry ice to the Institute of Clinical Molecular Biology at the Christian-Albrechts-University Kiel, Germany, for further processing. The V3V4 region of the 16S rRNA gene was sequenced using 341F (5′-CCTACGGGAGGCAGCAG-3′) and 806R (5′-GGACTACHVGGGTWTCTAAT-3′) as primers following the method description published in Rausch et al., 2019 [40]. Archaea were sequenced after a nested PCR approach using 344F (ACGGGGYGCAGCAGGCGCGA) and 1041R (GGCCATGCACCWCCTCTC) as first primer set and 519F (CAGCMGCCGCGGTAA) and 806R (GGACTACVSGGGTATCTAAT) as second primer set as described in Pausan et al. [41], and von Hoyningen-Huene et al. [42]. Extraction blanks and negative controls were included throughout the DNA extraction procedure to monitor potential contamination associated with sample processing. The DADA2 pipeline [43] for modeling and correcting Illumina-sequenced amplicon errors for quality-filtering was used with standard settings as implemented in the QIIME2 2023.2 microbiome bioinformatics platform [44,45]. QIIME2 was integrated in an own non-public instance of Galaxy (MedBionode https://usegalaxy.medunigraz.at (accessed on 3 April 2026)). Taxonomic assignment of the DADA2 representative sequences was obtained using the QIIME2 sklearn-based classifier for the SILVA rRNA database release 138 at 99% identity [46] for microbiome and archaeome. The sequences of the three different stool sample types were analyzed separately for Bacteria and Archaea using the MicrobiomeAnalyst 2.0 software [47,48]. Microbiome analyses were performed to characterize within-sample diversity, between-sample compositional differences, taxonomic discriminants, and community co-occurrence structure across anatomical compartments. Alpha diversity was assessed using the Chao1, Shannon, Simpson, Fisher, and observed features indices to capture richness and diversity-related properties of bacterial and archaeal communities. Statistically significant variations among the study groups were evaluated using a one-way analysis of variance (ANOVA), followed by Welch *T* Test. Beta diversity was evaluated across colon, ileum, and stool using Aitchison distances and global PERMANOVA, with pairwise comparison matrices reported in Supplementary Table S1.

Differentially abundant taxa were exploratively identified using linear discriminant analysis effect size (LEfSe; MicrobiomeAnalyst 2.0 software ) with a significance threshold of *p* < 0.1 and an LDA score > 2.0. To assess whether tumor-associated dysbiosis reflected coordinated community shifts or isolated taxonomic changes, sparsity-aware co-occurrence networks were constructed at the genus level using the SECOM approach, applying a correlation threshold > 0.3 and *p* < 0.05. Network structure was compared across bacterial and archaeal domains and anatomical compartments.

### 2.7. Statistical Analysis

Data was managed with Microsoft Excel 2016^®^ spreadsheets. GraphPad^®^ 10 (GraphPad Software Inc., Boston, MA, USA) was used for statistical analysis. Metric data is displayed as mean and standard deviation. The non-parametric Kruskal–Wallis test followed by a Dunn’s post hoc test was used for more than two groups and the Mann–Whitney test was used for comparing two groups. Gene Set Enrichment Analysis (GSEA) [49] was conducted as pairwise comparisons via gene lists ranked by WALD statistics using the GSEA desktop application and the Hallmark gene set list of the Molecular Signatures Database (MSigDB) [50]. Single Sample Gene Set Enrichment Analysis (ssGSEA) [51] was calculated to assess how tumor topography affects the host in the absence of an adaptive T-cell compartment in R using RStudio 2026.04.0+526 and the packages msigdbr, pheatmap, ggplot2, dplyr, tidyr, tibble, igraph, forcats, GSVA, GSEABase and limma. Data Integration Analysis for Biomarker Discovery using Latent Variable Approaches for Omics Studies (DIABLO) [52,53] was also calculated in R using RStudio 2026.04.0+526 and the packages mixOmics, dplyr, stringer, writexl, reshape2, igraph, ggraph, tidyr, gprofiler2 and ggplot2. Informative features were defined as the top 15 by frequency and top 15 by the strongest impact on group separation within the integrated dataset. Predictive performance of the trained DIABLO model was evaluated using cumulative receiver operating characteristic (ROC) analyses based on Components 1–2 across the primary feature blocks. Discrimination between experimental groups was quantified by the area under the ROC curve (AUC). Pathway enrichment was conducted on DIABLO loadings–weighted transcriptomic features from each block using Hallmark gene sets. We then generated pathway profiles for each host tissue and the human xenograft compartment. *p*-values ≤ 0.05 were considered statistically significant labelled as *p* ≤ 0.05 *, *p* ≤ 0.01 **, *p* ≤ 0.001 *** and *p* ≤ 0.0001 ****. The numbers of biological replicates included in each analysis are detailed in Supplementary Table S6.

## 3. Results

All 12 animals injected with HuH6 cells developed a tumor, with one animal dying from the orthotopic group before tissue sampling due to unknown circumstances. After an average tumor growth of 11.4 weeks the animals were euthanized with an average tumor weight of 1.3 ± 1.0 g for the orthotopic group (5.5 ± 4.6% of the tumor-free bodyweight) and 1.6 ± 0.2 g for the ectopic group (6.7 ± 0.9% of the tumor-free bodyweight). The organ weight did not differ significantly between the groups except for the weight of the inguinal white adipose tissue (WAT) and the total WAT that was significantly lower in the ectopic vs. the control group (Table 1).

**Figure 1:**
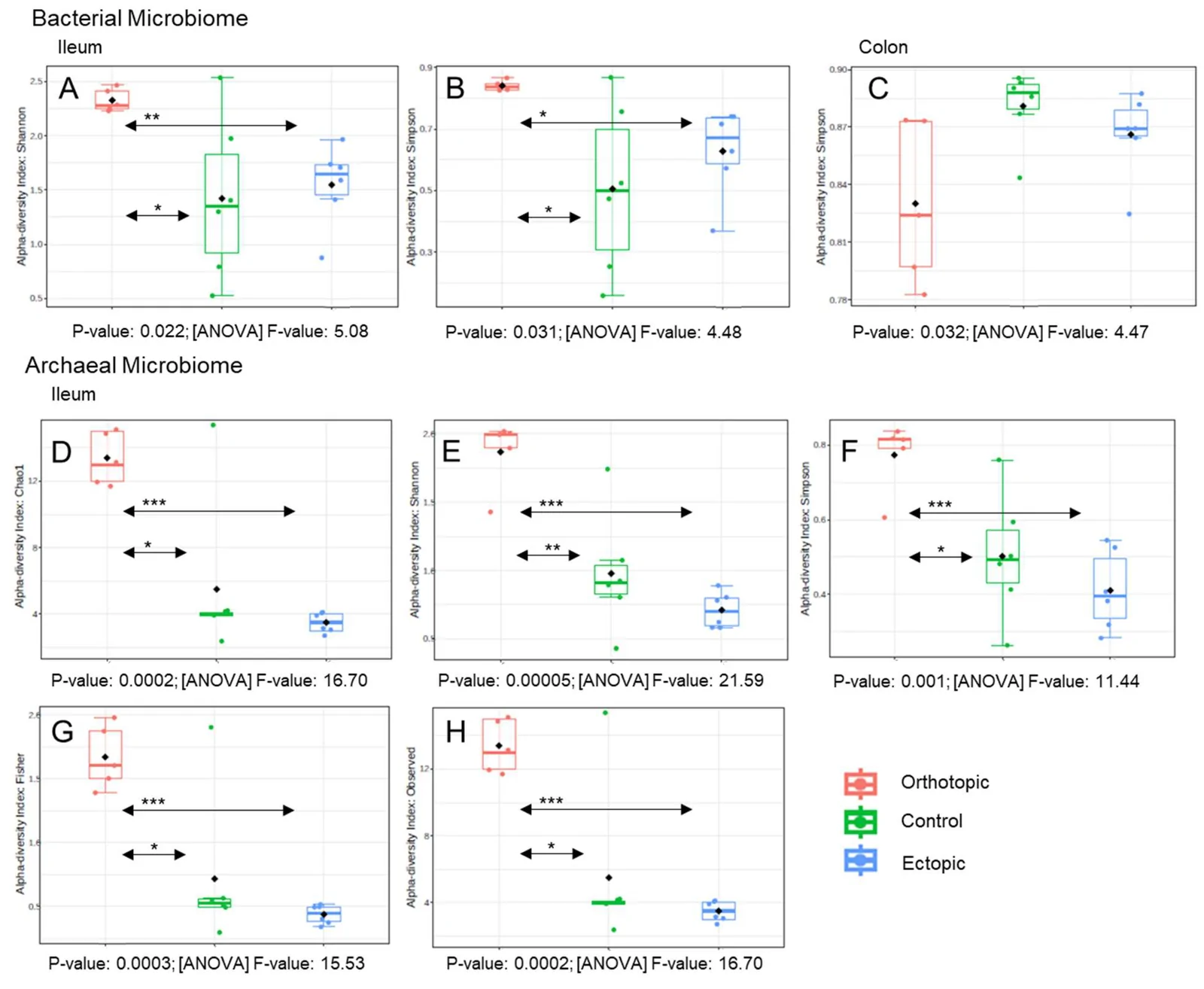
Significant alpha diversity patterns comparing tissue samples (*p* < 0.05). Alpha diversity of the bacterial (**A**–**C**) and archaeal (**D**–**H**) microbiome was assessed using the Shannon (**A**,**E**), Simpson (**B**,**C**,**F**), Chao1 (**D**), Fisher (**G**), and Observed (**H**) feature indices across the three experimental groups in Ileum (**A**,**B**,**D**–**H**) and Colon (**C**). The global differences between groups in all sampling sites were evaluated using a one-way ANOVA followed by a Welch *t*-test. Black diamonds indicate group means, and horizontal lines represent medians. orthotopic *n* = 5, ectopic *n* = 6, control *n* = 6. Arrows indicate groups with significant differences. *p* ≤ 0.05 *, *p* ≤ 0.01 **, *p* ≤ 0.001 ***.

Bacterial alpha diversity differed among the three groups for both the Shannon and Simpson indices (ANOVA, *p* < 0.05) in ileum and the Simpson index in colon (*p* = 0.032) with a trend in the Shannon index (*p* = 0.084, F = 2.97) (Figure 1A–C). Stool samples showed no differences. Subsequent pairwise comparisons of the significantly different alpha diversities demonstrated that orthotopic tumor samples (*n* = 5) had significantly higher diversity compared to both the control (*n* = 6) and ectopic (*n* = 6) tumor groups (FDR < 0.05). Archaeal alpha diversity (Figure 1D–H) revealed no significant differences in the stool samples. The colon sample evaluation displayed only a trend in Simpson’s index (*p* = 0.089, F = 2.89). In ileum samples, however, all five measured indices differed significantly between the three groups. Post-hoc analysis revealed significantly higher values in the orthotopic group compared to both the control and ectopic groups.

### 3.1. Compartment-Specific Differences in Bacterial and Archaeal Beta Diversity in Response to Tumor Topography

**Figure 2:**
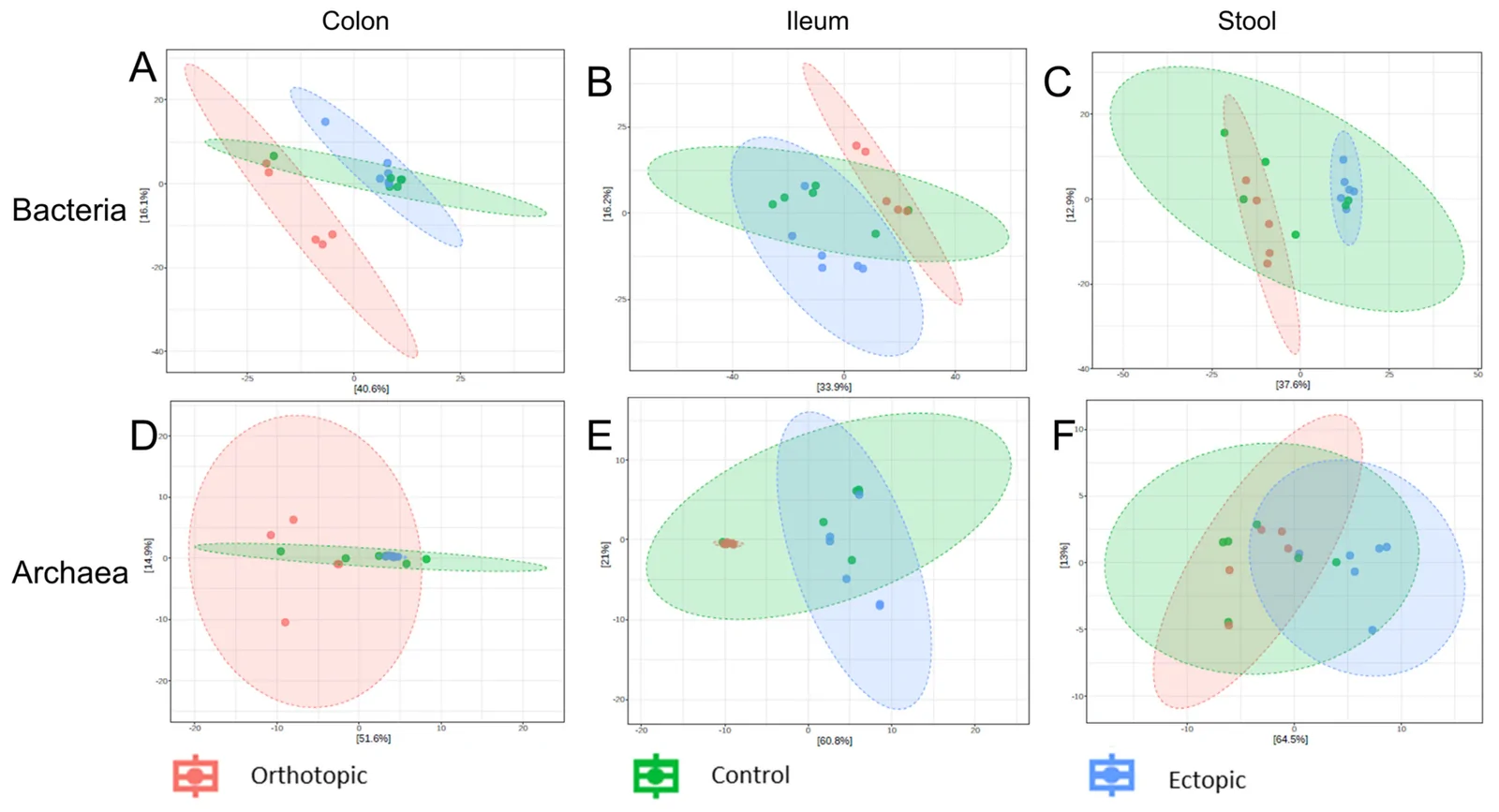
Compartment-specific shifts in bacterial and archaeal beta diversity across tumor topographies. Principal coordinate analysis (PCoA) based on Aitchison distances shows microbial community structure for bacteria (**A**–**C**) and archaea (**D**–**F**) in colon (**A**,**D**), ileum (**B**,**E**), and stool (**C**,**F**). Ellipses indicate 95% confidence intervals. Global differences were assessed by PERMANOVA (9999 permutations); the strength and significance of group separation varied by compartment and microbial domain. Detailed R^2^, F, and *p* values are provided in the main text and Supplementary Table S1. orthotopic *n* = 5, ectopic *n* = 6, control *n* = 6.

Within the bacterial domain, structural shifts in the ileum and colon were found which were not detectable in stool samples. In the colon, community composition was significantly altered by both oncogenic conditions, with all groups diverging from one another (global: F = 2.86, R^2^ = 0.290, *p* = 0.006; Figure 2A). Pairwise post-hoc comparisons confirmed significant differences for orthotopic vs. control (FDR = 0.0225), orthotopic vs. ectopic (FDR = 0.0225), and control vs. ectopic (FDR = 0.004). In contrast, the bacterial community in the ileum (Figure 2B) demonstrated a distinct susceptibility to hepatic malignancy (global: F = 4.74, R^2^ = 0.404, *p* = 0.013). Pairwise testing demonstrated that while the microbiota in the orthotopic group significantly diverged from both control (FDR = 0.0225) and ectopic tumor samples (FDR = 0.009, R^2^ = 0.504), subcutaneous tumors induced no detectable shift relative to healthy controls (FDR = 0.321). Strikingly, this tumor-driven deviation of the bacterial community did not translate to the fecal niche, as stool bacterial beta diversity (Figure 2C) remained statistically indistinguishable among all groups (F = 1.66, R^2^ = 0.192, *p* = 0.123).

The archaeal domain revealed a more pronounced, binary separation between tumor models that sharply contrasted between ileum content (Figure 2D) and colon content (Figure 2E) and the stool samples (Figure 2F). In samples of colon and ileum, archaeal communities were exclusively perturbed by the presence of liver tumors. In the colon (global: F = 4.54, R^2^ = 0.393, *p* = 0.005), the orthotopic group significantly differed from control (FDR = 0.0165) and ectopic (FDR = 0.0150), whereas ectopic remained indistinguishable from control (FDR = 0.982). This hepatic tumor-specific effect was maximized in the ileum (global: F = 9.81, R^2^ = 0.583, *p* = 0.001), where the model factor accounted for 70.2% of the variation in the distance matrix compared to subcutaneous tumors (orthotopic vs. ectopic: FDR = 0.0150; orthotopic vs. control: FDR = 0.0165), while ectopic tumors showed no deviation from control (FDR = 0.390). Remarkably, this finding completely inverted within the stool niche, showing an effect unique to the archaeal kingdom. Stool archaeal beta diversity differed by group, (global: F = 12.12, R^2^ = 0.634, *p* = 0.002), but this effect was entirely driven by the ectopic model. Post-hoc analysis of stool samples revealed that the ectopic group significantly diverged from both the control (FDR = 0.027, R^2^ = 0.490) and orthotopic groups (FDR = 0.015, R^2^ = 0.690). Conversely, stool archaeal composition in the orthotopic group did not differ significantly from that of healthy controls (FDR = 0.429). Taken together, these data indicate compartment-specific microbiome alterations associated with tumor localization, with orthotopic HB primarily linked to bacterial and archaeal shifts, whereas stool archaeal changes were mainly observed in the ectopic model (for pairwise comparison matrices see Supplementary Table S1).

### 3.2. Exploratory Analysis of Taxonomic Profiles Reveals Distinct Patterns Across Anatomical Niches

**Figure 3:**
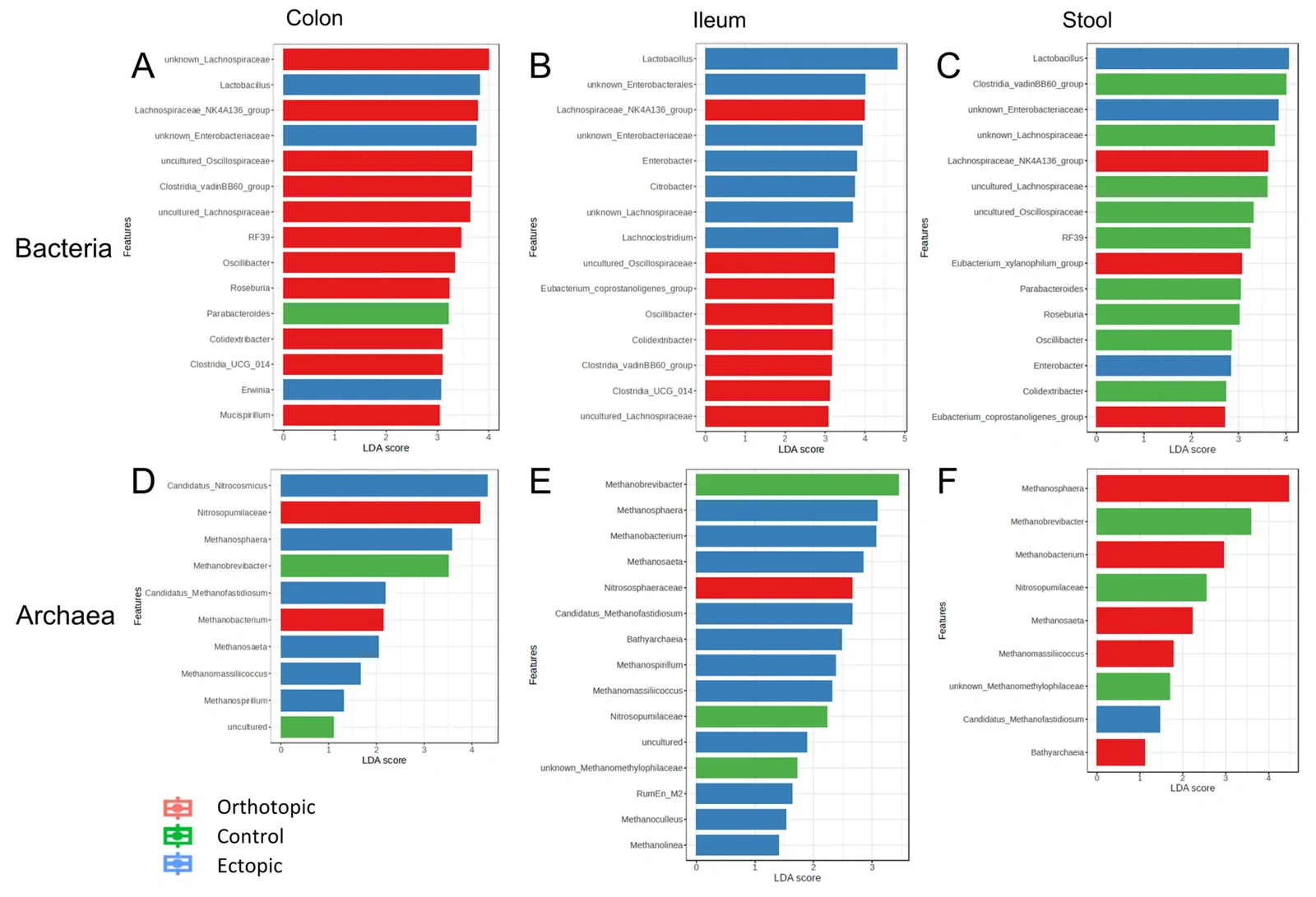
Linear discriminant analysis effect size (LefSe) shows niche- and domain-specific discriminative microbial taxa. Bar plots show genus-level features with discriminatory power (*p* < 0.1, LDA score > 2.0) for bacteria (**A**–**C**) and archaea (**D**–**F**) across the colon (**A**,**D**), ileum (**B**,**E**), and stool (**C**,**F**). Taxa are color-coded according to the experimental group in which they are most abundant. Ileum and colon content (**A**,**B**,**D**,**E**) show group-specific taxonomic enrichment patterns, whereas the fecal archaeal niche (**F**) is predominantly associated with the ectopic tumor model. LefSe biomarker profiles see Supplementary Table S2. orthotopic *n* = 5, ectopic *n* = 6, control *n* = 6.

Bacterial LEfSe analysis of the colon (Figure 3A) showed loss of homeostatic taxa in tumor-bearing mice, while controls retained short-chain fatty acid (SCFA) producers, including *Roseburia*, *Oscillibacter*, and *Lachnospiraceae*. Orthotopic tumors were enriched for *Lactobacillus* and *Enterobacteriaceae* while ectopic tumors were enriched for *Parabacteroides*. In the ileum (Figure 3B), ectopic samples remained control-like, dominated by *Lactobacillus* and *Enterobacter*, whereas orthotopic samples correlated with colonic anaerobes such as *Oscillibacter* and *Clostridia*. In stool, this bacterial signature was attenuated, consistent with the lack of global beta-diversity significance (Figure 3C), though feces from the ectopic tumor group retained residual enrichment of diverse *Lachnospiraceae* species.

Archaeal LEfSe analysis revealed compartment-specific patterns between intestinal contents and stool. In colon (Figure 3D) and ileum (Figure 3E) contents, methanogenic and ammonia-oxidizing taxa showed differential abundance across the hepatic malignancy groups. In the colon, the orthotopic phenotype was driven by the ammonia-oxidizer Nitrosopumilaceae and *Methanobacterium*, whereas the ectopic group was characterized by a consortium including *Candidatus Nitrocosmicus*, *Methanosphaera*, and *Candidatus Methanofastidiosum*. Effects were strongest in the ileum, a niche atypical for complex archaeal communities: Ectopic samples dominated the profile, expanding strictly anaerobic, hydrogen- and acetate-utilizing genera (*Methanosphaera*, *Methanobacterium*, *Methanosaeta*, *Bathyarchaeia*, *Methanospirillum*), whereas orthotopic samples showed a solitary enrichment of Nitrosospheraceae.

This pattern inverted in stool material. Stool archaeal signatures (Figure 3F) in ectopic mice were markedly reduced, yielding virtually no discriminative features aside from minor enrichment of *Candidatus Methanofastidiosum*. In contrast, the orthotopic model captured the stool archaeal niche with expansion of *Methanosphaera*, *Methanobacterium*, and *Methanosaeta*, shifting homeostatic stool archaea (*Methanobrevibacter* and Nitrosopumilaceae) to healthy controls.

Collectively, content-associated archaeal profiles showed compartment-specific patterns, with differences between tumor models being more evident in orthotopic HB models, whereas stool archaeal profiles were model-specific and did not consistently mirror the patterns observed in intestinal contents. This spatial compartmentalization provides a framework for integrating host transcriptomic data to further investigate tissue-specific host–microbiome associations. (biomarker profile in Supplementary Table S2).

### 3.3. Sparsity-Aware Networks Identify Domain- and Compartment-Specific Microbial Interaction Patterns

**Figure 4:**
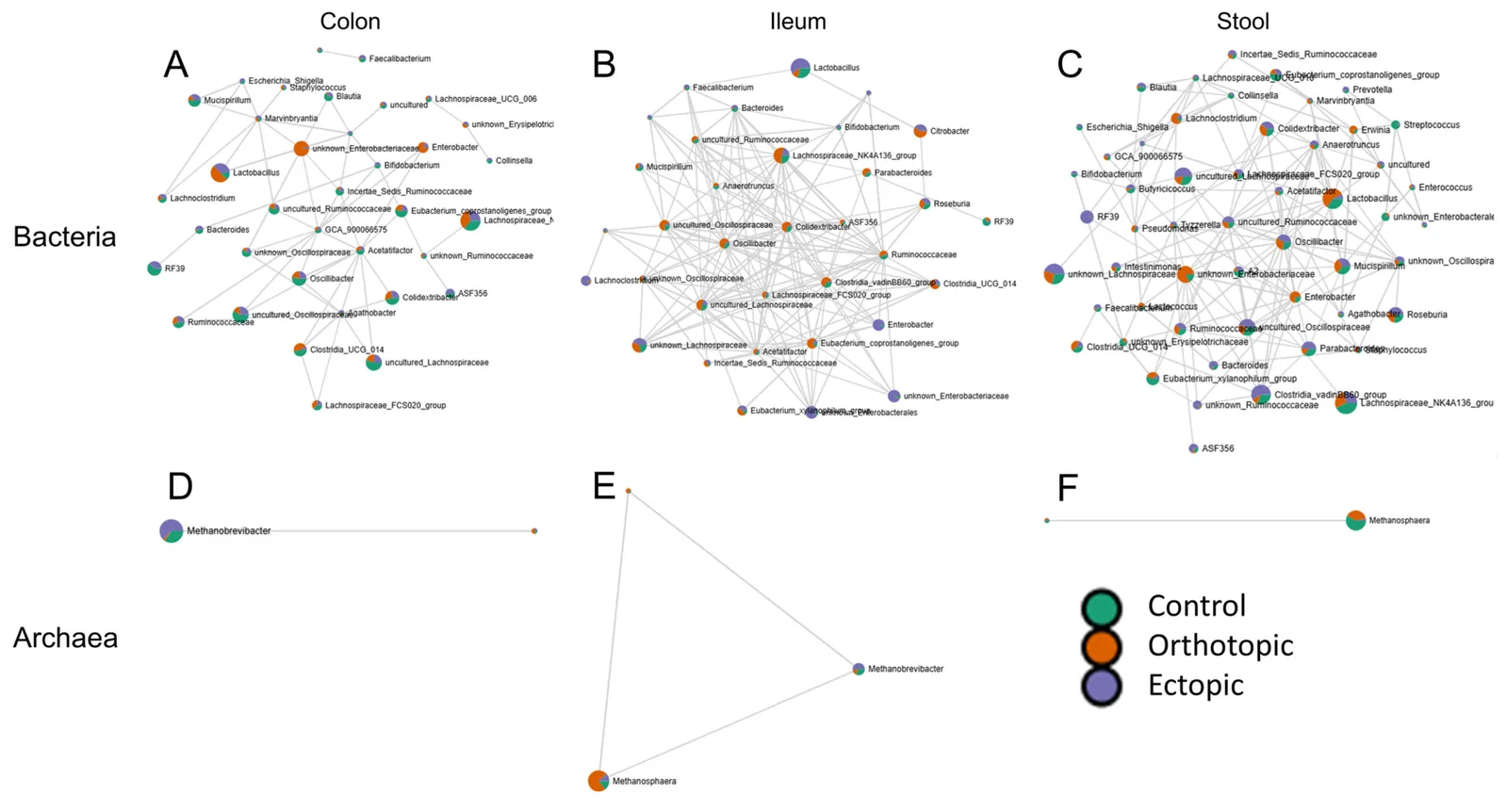
Sparsity-aware co-occurrence networks of bacterial and archaeal genera across anatomical niches. Network inference was performed using the SECOM algorithm (Pearson2; correlation > 0.3, *p* < 0.05) for bacteria (**A**–**C**) and archaea (**D**–**F**) in colon (**A**,**D**), ileum (**B**,**E**), and stool (**C**,**F**). Node size reflects relative abundance, and pie charts indicate mean group distribution. Bacterial networks show compartment-specific differences in connectivity, whereas archaeal networks are markedly sparser and dominated by a small number of taxa. orthotopic *n* = 5, ectopic *n* = 6, control *n* = 6.

Bacterial sparsity-aware co-occurrence network (Figure 4) complexity differed between intestinal segments and stool samples. In the colon (Figure 4A), the network showed distinct clustering of taxa, with one module comprising mainly taxa from the Oscillospiraceae and Lachnospiraceae families and another containing taxa enriched in the orthotopic HB group, including Enterobacteriaceae, *Enterobacter*, and *Escherichia_Shigella*. *Lactobacillus* occupied a connected position within this network. In the ileum (Figure 4B), connectivity was increased and the network was dominated by taxa associated with the orthotopic group, including *Lachnospiraceae_NK4A136_group*, *Oscillibacter*, and *Colidextribacter*. Taxa associated with the ectopic group and with control samples were positioned more peripherally. Stool samples’ networks (Figure 4C) were more fragmented and showed less distinct group-associated structure.

Archaeal networks were markedly sparser than in bacteria. In the colon (Figure 4D), connectivity was limited and centered on *Methanobrevibacter* and a low-abundance unclassified genus. In the ileum (Figure 4E), the network consisted of three nodes linked by positive associations, including *Methanosphaera*, *Methanobrevibacter*, and a low-abundance unclassified taxon. In stool (Figure 4F), interactions were again sparse and were primarily centered on *Methanosphaera*. Across compartments, network structures differed by tumor model and sample type, with the most pronounced differences observed in intestinal content samples.

Together, these networks indicate that tumor localization remodels microbial co-occurrence in a compartment-specific manner, yielding stronger, more complex patterns in intestinal segments than in stool, and a particularly sparse, compartment-dependent archaeal architecture.

### 3.4. Compartment-Specific Host Transcriptomic Response Is Characterized by Innate Immune Remodeling and Metabolic Stress

**Figure 5:**
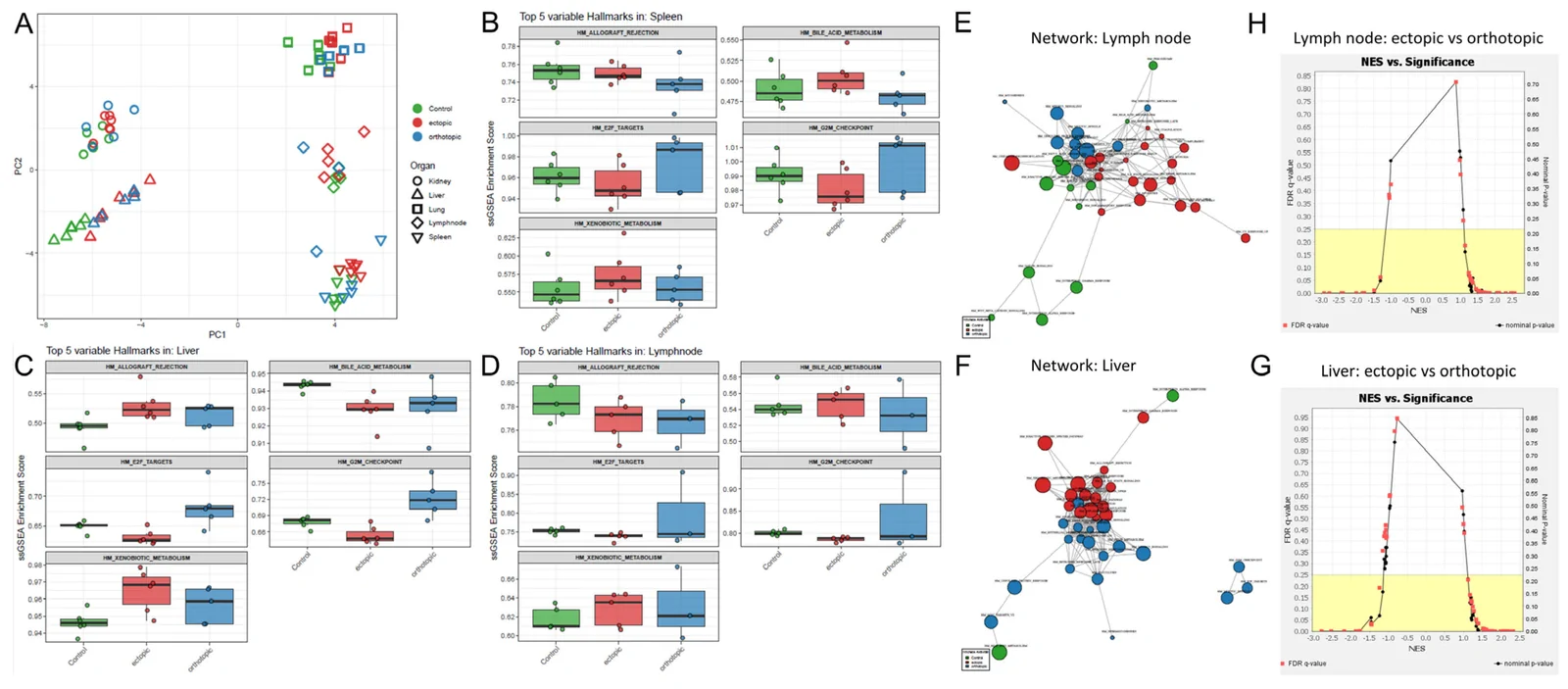
Compartment-specific host transcriptomic profiling reveals organ-dependent hallmark remodeling in athymic CAnN.Cg-Foxn1nu/Crl mice. Principal component analysis (**A**) of ssGSEA (single-sample Gene Set Enrichment Analysis) hallmark scores shows separation of samples by organ and tumor model. Boxplots (**B**–**D**) display the five most variable hallmarks in spleen (**B**), liver (**C**), and mesenteric lymph nodes (**D**). Network analyses (**E**,**F**) depict hallmark co-enrichment patterns in mesenteric lymph nodes and liver parenchyma, respectively. GSEA summary plots (**G**,**H**) show differential enrichment patterns in liver and mesenteric lymph nodes between orthotopic (blue) and ectopic (red) groups. Group sizes: kidney, lung, liver, and spleen tissues: orthotopic (*n* = 5), ectopic (*n* = 6), and control (*n* = 6); for lymph nodes: orthotopic (*n* = 3), ectopic (*n* = 5), and control (*n* = 5).

To test tumor topography effects without adaptive T-cells, we evaluated ssGSEA (heatmaps see Supplementary Figure S1) profiles across multiple murine organs in male athymic CAnN.Cg-Foxn1nu/Crl mice. Given the severe T-cell deficiency of our murine model, detected alterations in immune hallmarks mostly reflect remodeling of the innate immune system and myeloid lineages. In alignment with our multivariate Principal Component Analysis framework (PCA; Figure 5A), control compartments (kidney, lung) were transcriptomically stable with overlapping enrichment scores across all cohorts (control and ectopic *n* = 6, orthotopic *n* = 5 except lymph node: orthotopic *n* = 3; ectopic *n* = 5; control *n* = 5 for murine; ectopic *n* = 6 and orthotopic *n* = 4 for human) (Supplementary Figure S2).

In the lung, changes were limited and mainly involved reduced enrichment of proliferation-related hallmarks (HM_E2F_TARGETS and HM_G2M_CHECKPOINT) in ectopic tumor-bearing mice, while orthotopic tumors had little effect on pulmonary hallmark profiles.

In mesenteric lymph nodes (Figure 5D), orthotopic tumors increased proliferation-related pathways in a subset of samples, indicating a topography-dependent lymphatic response. Controls and ectopic models remained quiescent, whereas a distinct orthotopic subset displayed upregulated cell-cycle hallmarks (HM_E2F_TARGETS and HM_G2M_CHECKPOINT). In the liver (Figure 5C), both tumor models altered immune and metabolic hallmarks. HALLMARK_ALLOGRAFT_REJECTION and HALLMARK_XENOBIOTIC_METABOLISM were increased in both groups, while HALLMARK_BILE_ACID_METABOLISM was reduced. Proliferation-related hallmarks, including E2F_TARGETS and G2M_CHECKPOINT, were more strongly enriched in the orthotopic group.

Spleen profiling (Figure 5B) showed opposing patterns. Ectopic tumors were associated with reduced enrichment of E2F_TARGETS, G2M_CHECKPOINT, and MYC_TARGETS_V1, whereas Orthotopic tumors showed increased enrichment of these proliferation-related hallmarks. Differences also involved HALLMARK_HEME_METABOLISM and HALLMARK_INFLAMMATORY_RESPONSE. Overall, these data indicate that tumor localization is associated with organ-specific transcriptional remodeling in the host, with the strongest effects in secondary lymphoid organs and liver tissue in this athymic mouse model.

### 3.5. Topological Hallmark Networks Reveal Compartment-Dependent Coordination Patterns

In the mesenteric lymph nodes (Figure 5E), the co-enrichment network separated proliferation-related hallmarks associated with the Orthotopic group and stress- and inflammation-related hallmarks associated with the Ectopic group. Orthotopic-associated hallmarks included E2F_TARGETS, G2M_CHECKPOINT, and MYC_TARGETS_V1, whereas Ectopic-associated hallmarks were enriched for HALLMARK_TNFA_SIGNALING_VIA_NFKB, HALLMARK_HYPOXIA, and HALLMARK_APOPTOSIS. Homeostatic programs localized centrally, while interferon pathways were peripheral.

In the liver (Figure 5F), the co-enrichment network showed a clear separation between a proliferation-related module (HM_E2F_TARGETS, HM_G2M_CHECKPOINT, and HM_MITOTIC_SPINDLE) and a larger host-response module. Proliferation-related hallmarks associated with the Orthotopic group formed a compact cluster, while xenobiotic metabolism, hypoxia, and glycolysis were associated with the host response and showed stronger connections within the main network core. These patterns are consistent with compartment-specific remodeling of host transcriptional programs in response to tumor topography.

### 3.6. Global GSEA Summaries Support Topography-Dependent Patterns

Global GSEA summary plots showed that murine liver (Figure 5G) and murine mesenteric lymph node (Figure 5H) profiles differed between orthotopic and ectopic groups, indicating organ-specific pathway remodeling.

In the liver, xenobiotic metabolism and related stress-response pathways were strongly represented. While baseline maintenance pathways remained uniform, the interactome showed a thermodynamic extreme across the negative operational margin, with Normalized Enrichment Scores (NESs) dropping down to <−3.0.

The mesenteric lymph node (Figure 5H) showed more pronounced enrichment of proliferation- and immune-related hallmarks. This was defined by a robust accumulation of significant functional modules shifting exclusively toward the positive axis (NES > 1.2 at FDR q-value = 0.00). Together, these analyses indicate that tumor topography shapes host transcriptional responses in a compartment-dependent manner.

**Figure 6:**
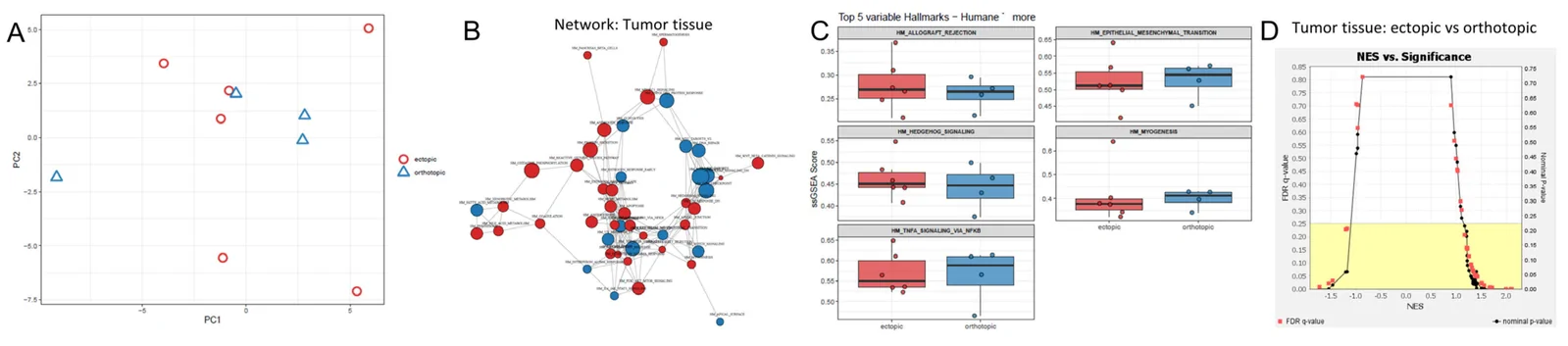
The hepatic microenvironment shapes the human xenograft transcriptome toward more coordinated hallmark profiles. (**A**) Principal component analysis of human ssGSEA hallmark scores comparing ectopic subcutaneous tumors and orthotopic liver tumors. (**B**) Sparsity-aware hallmark co-regulatory network of the human xenograft cells. Nodes represent individual hallmark pathways, node size reflects relative abundance, and colors indicate the group with the highest enrichment. Edges represent statistically significant correlations. (**C**) Boxplots showing the five most variable hallmarks between growth conditions, including HALLMARK_ALLOGRAFT_REJECTION, HALLMARK_EPITHELIAL_MESENCHYMAL_TRANSITION, HALLMARK_HEDGEHOG_SIGNALING, HALLMARK_MYOGENESIS, and HALLMARK_TNFA_SIGNALING_VIA_NFKB. (**D**) Global GSEA performance summary plot for the human xenograft cells comparing ectopic and orthotopic tumors. The plot shows a relatively narrow NES range and a strong concentration of significant hallmarks near the center of the distribution. Group size: orthotopic *n* = 4 and ectopic *n* = 6.

Global GSEA summary plots for the human xenograft cells confirmed a relatively concentrated distribution of significant pathways between orthotopic and ectopic conditions (Figure 6D). Differences were observed for hallmarks related to proliferation, epithelial–mesenchymal transition, inflammatory signaling, and metabolic adaptation. The overall NES range (FDR q-value = 0.00) was narrower than in the host compartments, suggesting a more focused transcriptional response to engraftment site in the human tumor than in surrounding murine tissues.

### 3.7. Multivariate Stabilization of the Human Xenograft Transcriptome Within the Hepatic Niche

We analyzed ssGSEA hallmark scores from the human xenograft tissues and compared orthotopic and ectopic growth conditions (Supplementary Table S3).

Principal Component Analysis (PCA) of these scores showed clear differences in intra-group variance (Figure 6A). Human tumor cells residing within their native, orthotopic microenvironment exhibit a more homogeneous transcriptional profile within the hepatic niche. In contrast, tumor cells expanding within the artificial subcutaneous niche (ectopic) showed broader dispersion along both major axes, consistent with greater transcriptional heterogeneity.

To examine pathway relationships, we constructed a sparsity-aware hallmark co-regulatory network (Figure 6B). The orthotopic group network was more compact, with proliferation-related hallmarks such as HALLMARK_E2F_TARGETS and HALLMARK_G2M_CHECKPOINT positioned close to HALLMARK_EPITHELIAL_MESENCHYMAL_TRANSITION. In contrast, ectopic group-associated hallmarks were more broadly distributed and included HALLMARK_OXIDATIVE_PHOSPHORYLATION and HALLMARK_XENOBIOTIC_METABOLISM.

Analysis of the most variable hallmarks supported these model-dependent differences (Figure 6C). HALLMARK_EPITHELIAL_MESENCHYMAL_TRANSITION showed higher scores in the orthotopic group, together with increased enrichment of HALLMARK_TNFA_SIGNALING_VIA_NFKB. HALLMARK_MYOGENESIS was among the variable hallmarks, whereas HALLMARK_HEDGEHOG_SIGNALING showed relatively similar scores across both groups. Overall, the hepatic niche was associated with a more coordinated proliferative and inflammatory hallmark profile in the human xenografts, whereas ectopic growth was associated with greater transcriptional dispersion.

### 3.8. Multi-Omics Integration Reveals Compartment-Dependent Correlation Patterns Across Host, Microbiome, and Tumor Blocks

**Figure 7:**
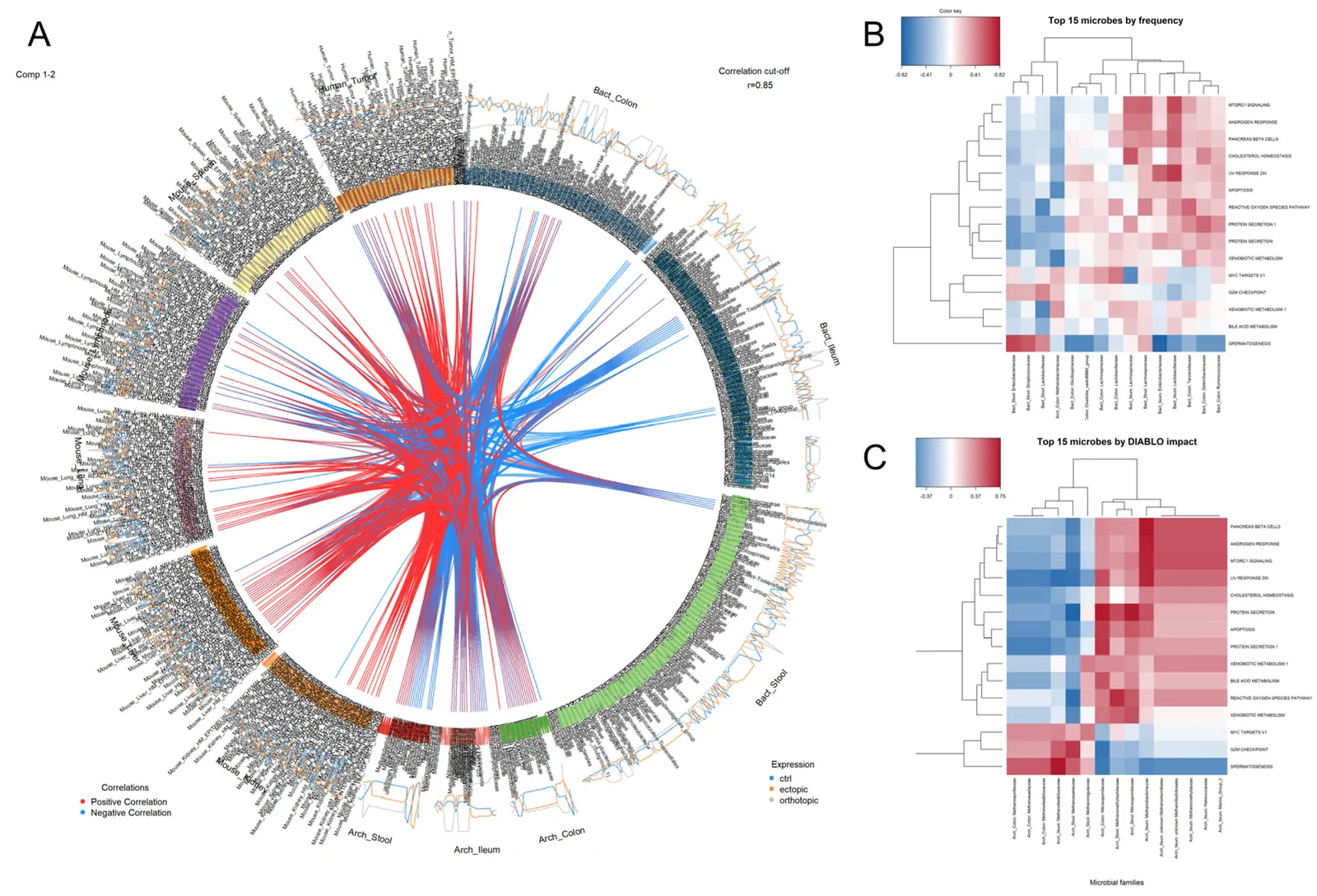
DIABLO multi-omics block integration identifies inter-block correlation patterns across host, microbiome, and tumor compartments. (**A**) Circos plot showing pairwise correlations between multi-omics data blocks on components 1 and 2, including the human tumor block (Human_Tumor), five murine host compartments, and bacterial and archaeal families from ileum, colon, and stool. Correlations with *r* ≥ 0.85 are shown. Outer tracks display mean feature values across control, ectopic, and orthotopic groups. (**B**,**C**) Hierarchical clustered Spearman correlation heatmaps showing the 15 microbial features most frequently selected across the DIABLO analysis (**B**) and the 15 microbial features with the highest absolute DIABLO loading values (**C**), together with the corresponding Hallmark features. Positive correlations are shown in red and negative correlations in blue. Group size: kidney, lung, liver, and spleen tissues: orthotopic (*n* = 5), ectopic (*n* = 6), and control (*n* = 6); for lymph nodes: orthotopic (*n* = 3), ectopic (*n* = 5), control (*n* = 5); and for tumor tissues: orthotopic (*n* = 4) and ectopic (*n* = 6).

We integrated murine (control *n* = 6, ectopic *n* = 6, orthotopic *n* = 5) and human (ectopic *n* = 6, orthotopic *n* = 4) host transcriptomic, microbial, and tumor-associated features using DIABLO-based multi-block integration. The analysis included bacterial and archaeal families from ileum, colon, and stool, murine Hallmark pathway scores from liver, spleen, lung, kidney, and mesenteric lymph nodes, and human tumor Hallmark scores (Supplementary Table S4). Using a correlation cutoff of *r* ≥ 0.85, the resulting circos plot revealed extensive positive and negative associations across microbial, host, and tumor-associated blocks (Figure 7A). Microbial features from different intestinal compartments were connected with transcriptional profiles of multiple host organs as well as with the human tumor block, indicating coordinated variation across anatomical compartments.

The integrated network showed associations spanning intestinal microbial communities and host transcriptional programs, including features derived from the spleen, liver, lung, kidney, and mesenteric lymph nodes. The human tumor block was also connected to both microbial and murine host features, highlighting its integration within the broader host–microbiome correlation structure. These patterns indicate coordinated inter-block associations across anatomical compartments rather than independent variation within individual data blocks.

### 3.9. Archaeal Families Are Prominent Features of the Integrated Correlation Structure

We evaluated the 15 microbial features most frequently selected across the DIABLO analysis and the 15 microbial features with the highest absolute DIABLO loading values, together with the corresponding Hallmark features, to characterize the strongest host–microbiome associations. In the frequency-based heatmap (Figure 7B), archaeal families showed prominent correlations with host Hallmark features, including cell-cycle-related signatures such as E2F_TARGETS and G2M_CHECKPOINT. Additional associations involved Hallmark features from mesenteric lymph nodes and the human tumor block, including proliferation- and immune-related pathways.

The impact-based heatmap (Figure 7C) likewise showed several archaeal families among the microbial features with strong DIABLO loadings. Their associations extended to tumor-associated Hallmark features, including ANGIOGENESIS and MTORC1_SIGNALING, as well as interferon-related, metabolic, and stress-response pathways in host tissues. Together, these findings identify archaeal and bacterial features that are strongly associated with coordinated host and tumor transcriptional programs and nominate archaeal taxa as candidates for further mechanistic investigation.

### 3.10. Cumulative ROC Analysis Characterizes Classification Performance Across Selected Multi-Omics Blocks

**Figure 8:**
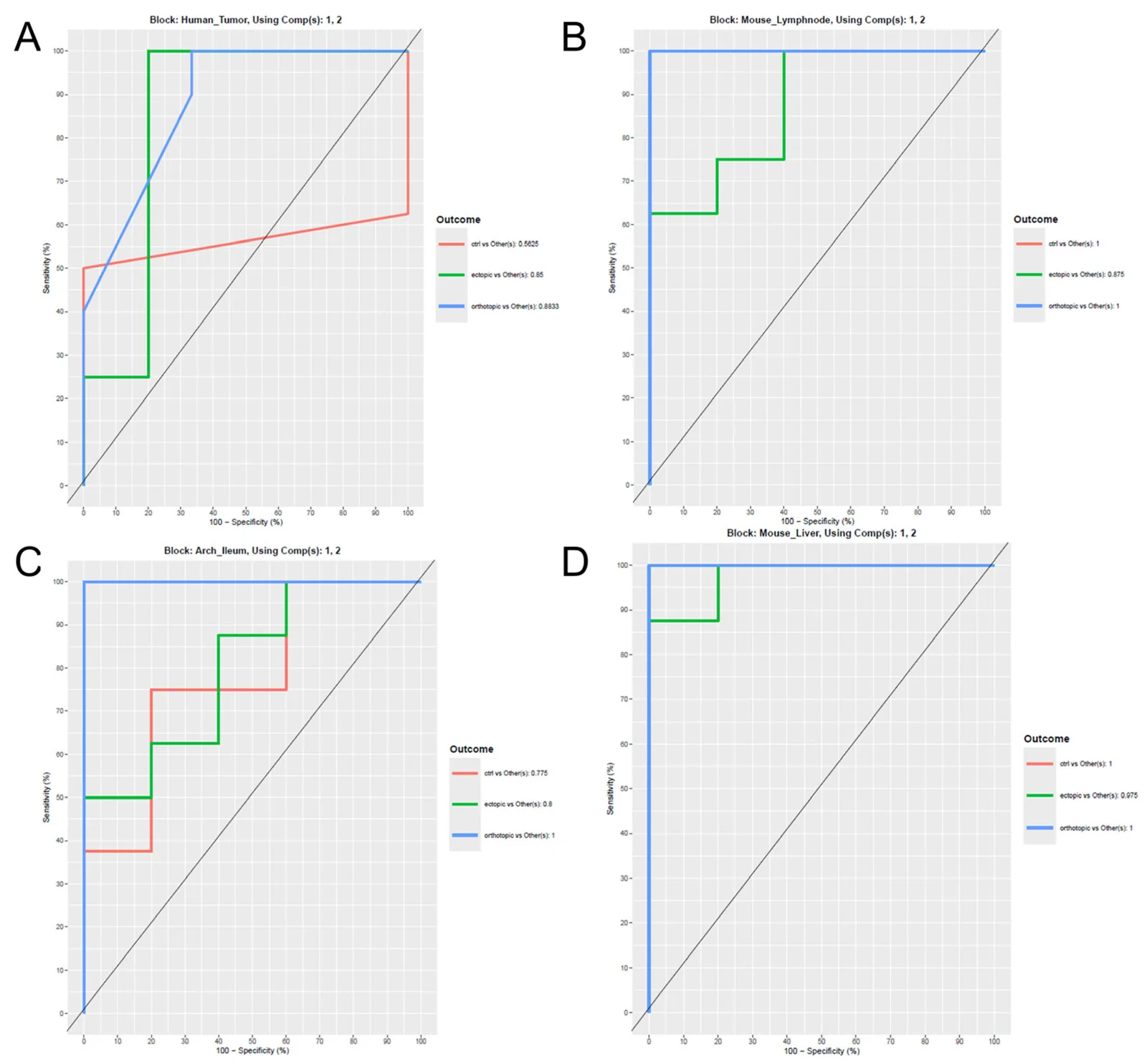
Cumulative ROC analysis characterizes the classification performance of the DIABLO model across selected host, microbial, and tumor-associated blocks. (**A**–**D**) Receiver operating characteristic (ROC) curves for the human tumor block (**A**), host mesenteric lymph nodes (**B**), archaeal ileum block (Arch_Ileum) (**C**), and host liver block (**D**). Curves represent the three outcome classes (control, ectopic, and orthotopic), with corresponding AUC values indicated in the legends. Classification performance was assessed using the cumulative contribution of Components 1 and 2. Group size: liver: orthotopic (*n* = 5), ectopic (*n* = 6), control (*n* = 6); lymph nodes: orthotopic (*n* = 3), ectopic (*n* = 5), control (*n* = 5); tumor tissues: orthotopic (*n* = 4) ectopic (*n* = 6); and archaeal microbiome: orthotopic (*n* = 5), ectopic (*n* = 6), control (*n* = 6).

Cumulative receiver operating characteristic (ROC) analysis was used to characterize the discriminatory performance of the DIABLO components across selected data blocks using the cumulative contribution of Components 1 and 2 (Figure 8). To further assess classification performance under repeated resampling, the two-component model was evaluated using repeated 5-fold cross-validation with 100 repeats, yielding an overall error rate of 9.8% and a balanced error rate of 13.5% (Table 2 and Table 3). Within the human tumor block, the cumulative model yielded AUC values of 0.56 for controls, 0.85 for the ectopic group, and 0.88 for the orthotopic group (Figure 8A). In the mesenteric lymph node block, AUC values were 1.00 for controls, 0.88 for the ectopic group, and 1.00 for the orthotopic group (Figure 8B).

The archaeal ileum block showed AUC values of 0.78, 0.80, and 1.00 for the control, ectopic, and orthotopic groups, respectively (Figure 8C). In the Mouse_Liver block, the corresponding AUC values were 1.00, 0.98, and 1.00 (Figure 8D).

Overall, the cumulative ROC analysis based on Components 1 and 2 showed variable discriminatory performance across data blocks and outcome groups, with particularly high AUC values for the orthotopic group in the human tumor and archaeal ileum blocks and for several classes in the lymph node and liver blocks. These results support the discriminatory structure identified by the DIABLO analysis, while the extent to which these performance estimates generalize beyond the analyzed cohort was further evaluated using repeated cross-validation.

### 3.11. DIABLO Model Weights Highlight Distinct Hallmark Signatures Across Host Tissues and Xenograft Tumors

**Figure 9:**
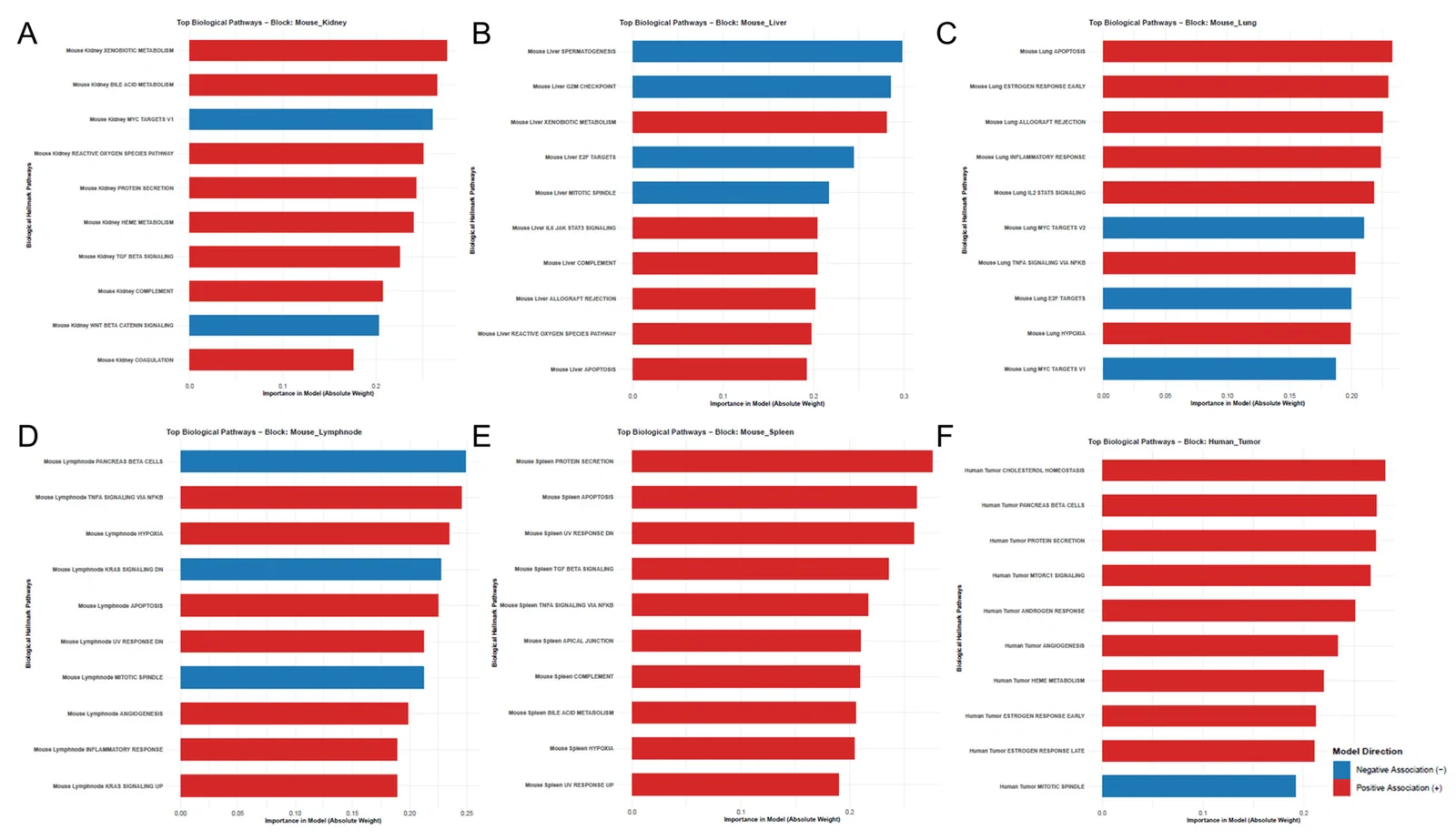
DIABLO model weights identify compartment-specific Hallmark features across host tissues and xenograft tumors. (**A**–**F**) Bar plots showing the ten Hallmark features with the highest absolute DIABLO model weights for murine kidney (**A**), liver (**B**), lung (**C**), mesenteric lymph nodes (**D**), spleen (**E**), and human xenograft tumor tissue (**F**). Bar length represents the absolute model weight, whereas color indicates the direction of the association (positive, red; negative, blue). The selected features differed across data blocks, highlighting compartment-specific transcriptional signatures contributing to the integrated DIABLO classification model. Group size: kidney, lung, liver, and spleen tissues: orthotopic (*n* = 5), ectopic (*n* = 6), and control (*n* = 6); for lymph nodes: orthotopic (*n* = 3), ectopic (*n* = 5), control (*n* = 5); and for tumor tissues: orthotopic (*n* = 4) and ectopic (*n* = 6).

To characterize the biological features contributing to the multiblock classification model, we examined the ten Hallmark features with the highest absolute DIABLO weights within each data block (Figure 9). The selected features differed substantially between host compartments, indicating compartment-specific transcriptional signatures associated with the multiblock model.

In the murine kidney (Figure 9A), the highest-weighted features included xenobiotic metabolism, bile acid metabolism, reactive oxygen species pathway, protein secretion, heme metabolism, and TGF-β signaling, with both positive and negative model associations represented. The liver block (Figure 9B) showed a distinct pattern that included spermatogenesis, G2M checkpoint, xenobiotic metabolism, E2F targets, mitotic spindle, IL6/JAK/STAT3 signaling, complement, allograft rejection, reactive oxygen species pathway, and apoptosis.

The lung block (Figure 9C) was characterized predominantly by positive model associations among the highest-weighted features, including apoptosis, estrogen response, allograft rejection, inflammatory response, IL6/JAK/STAT3 signaling, TNFα signaling via NF-κB, E2F targets, and interferon-α response, whereas MYC target signatures showed negative associations.

The mesenteric lymph node block (Figure 9D) showed a mixture of positive and negative associations. Among the highest-weighted features were TNFα signaling via NF-κB, apoptosis, inflammatory response, angiogenesis, and KRAS signaling, together with negatively associated pancreatic β-cell, KRAS signaling, IL6/JAK/STAT3, interferon-α, and mTORC1-related signatures.

The spleen block (Figure 9E) was dominated by positive associations among the highest-weighted features, including protein secretion, apoptosis, allograft rejection, TGF-β signaling, TNFα signaling via NF-κB, apical junction, complement, bile acid metabolism, hypoxia, and UV response.

The human tumor block (Figure 9F) likewise showed predominantly positive model associations among its highest-weighted features, with cholesterol homeostasis, pancreatic β-cell signatures, protein secretion, mTORC1 signaling, androgen response, angiogenesis, and additional metabolic and cell-cycle-related Hallmark features among the highest-weighted pathways.

Overall, the DIABLO model assigned distinct Hallmark features to individual host and tumor blocks, with differences in both pathway identity and direction of association across compartments. These patterns highlight compartment-specific transcriptional features contributing to the integrated classification structure rather than indicating independent pathway activity or mechanistic regulation.

## 4. Discussion

In this study, we investigated HB (HB) as a multi-compartment biological system, integrating tumor-intrinsic signaling, host organ responses, and spatially resolved gut microbial ecosystems in an orthotopic and ectopic human HuH6 xenograft model. By combining human tumor transcriptomics with host organ profiling and compartment-specific microbiome analysis across the ileum, colon, and feces—including both bacterial and archaeal communities—we provide a systems-level perspective on HB that extends beyond canonical tumor-centric frameworks. Our findings suggest that HB is associated with developmental, metabolic, immune-related, and microbially linked processes across multiple compartments [4,8,12,13,14,15,20].

A central observation of this work is the persistence of a fetal-like transcriptional state within HB tumors, consistent with previous descriptions of HB as a developmental malignancy [3,12,14]. The prominence of Wnt/β-catenin-driven transcriptional programs, together with Hippo/YAP-associated signaling modules, reinforces the notion that a limited set of developmental pathways is consistent with a role in maintaining proliferative competence and limit hepatocytic differentiation [14,17,18,21].

Crucially, our multi-omics framework shows that these canonical, human-centric oncogenic circuits are embedded within a broader systemic context, in which host organs exhibit coordinated transcriptional changes [24,25,26]. Our global single-sample Gene Set Enrichment Analysis (ssGSEA) and multivariate PCA mapping further supported coordinated transcriptional differences across multiple host compartments, suggesting that tumor-associated transcriptional changes were not confined to the malignant tissue itself but is accompanied by distinct transcriptional changes across host tissues [49,50].

The liver parenchyma, as the primary site of tumor growth in orthotopic settings, exhibited transcriptional signatures consistent with substantial host tissue remodeling [28,29,30]. Topological network reconstruction of the host liver interactome revealed a distinct bimodal separation. When colonized by orthotopic hepatic tumors, the murine liver parenchyma exhibited transcriptional features consistent with local transcriptional changes, including increased enrichment of tissue hypoxia (HM_HYPOXIA) and an enrichment of glycolysis-associated programs (HM_GLYCOLYSIS) [50]. This localized transcriptional changes were accompanied by an altered immune-related transcriptional profile, including positive enrichment of the HALLMARK_ALLOGRAFT_REJECTION pathway [14].

In ectopic tumor settings, these intrahepatic proliferative signatures were not observed, whereas systemic metabolic cues of the hepatic niche remained detectable. In the absence of local tumor colonization, distant subcutaneous tumor growth was associated with a prominent hepatic detoxification and oxidative stress signature, characterized by HM_XENOBIOTIC_METABOLISM [27,28]. Together, these findings suggest that HB is associated with topography-dependent host remodeling, with distinct liver-associated transcriptional patterns in orthotopic and ectopic settings [24,25].

Importantly, our data support a model of potential, anatomically constrained host–tumor–microbiome interactions rather than a unidirectional relationship between tumor growth and microbial remodeling [29,54]. While tumor presence is associated with microbiome remodeling, microbial ecosystems may be associated with tumor growth and host organ responses through potential metabolic and immunological interactions [29,54,55]. These observations align with emerging concepts in cancer systems biology, in which tumors are embedded within dynamic host networks rather than operating independently of the host [24,25,54]. In HB, these interactions may be particularly relevant given the anatomical coupling of the intestine and liver via the portal circulation [27,28,29,30].

Additional support for coordinated host–tumor–microbiome interactions was provided by the integrated DIABLO framework. Operating at an exceptionally stringent correlation threshold (correlation cut off: r = 0.85), the global circos interactome identified a highly connected pattern of associations linking tissue-associated intestinal microbial families with the host secondary lymphoid organs and the human xenograft itself [52,53].

Notably, the inter-domain interactome included substantial contributions from the non-bacterial ileum and colon domains [56,57,58]. Methanogenic archaea within the ileum content (Arch_Ileum) showed complete classification performance for the orthotopic phenotype (AUC = 1.0000), consistent with an association between their enrichment and the orthotopic model rather than reflecting overall disease burden alone [56,57].

The distribution of DIABLO model weights across host and tumor blocks further highlighted tissue-specific transcriptional signatures within the integrated model [52]. Across the analysed host tissues, highly weighted Hallmark features included pathways related to metabolism, cellular stress, inflammatory signaling, apoptosis, and developmental processes, with distinct patterns observed between individual organs [59]. The human xenograft compartment showed prominent associations with metabolic, proliferative, signaling, and protein secretion-related Hallmark features. These patterns provide biological context for the features identified by the DIABLO framework but should not be interpreted as direct evidence of pathway activation or mechanistic regulation.

From a developmental perspective, the integrated host–microbiome structure is consistent with the involvement of conserved signaling pathways relevant to fetal liver identity and hepatoblastoma biology [8,27,28,55]. Wnt/β-catenin and Hippo/YAP signaling are established components of hepatoblastoma biology and may be influenced by the metabolic and inflammatory context of the tumor microenvironment [8,16,17,18,21,60,61,62]. In our analysis, archaeal families including *Nitrosopumilaceae* and *Methanomassiliicoccaceae* were associated with transcriptomic features related to mTORC1 signaling and angiogenesis in the human xenograft compartment. These associations identify archaeal features as candidates for further investigation within the gut–liver–tumor axis, but do not establish a direct functional relationship or directionality [3,12,27,28,41,42].

The heterogeneity observed between orthotopic and ectopic tumor models further emphasizes the importance of anatomical context when interpreting tumor–host–microbiome interactions [24,25,27]. Orthotopic and ectopic tumors were associated with distinct host transcriptional and microbial patterns, with the integrated analyses indicating broader cross-compartment associations in the orthotopic setting. In the ectopic model, hepatic responses were characterized particularly by xenobiotic metabolism, whereas several of the broader gut-associated and host transcriptional patterns observed in the orthotopic model were less prominent. These differences suggest that tumor location can influence the extent and composition of host–microbiome associations captured by the integrated model [27,28,29,30].

The anatomical positioning of the tumor may therefore influence communication along the gut–liver axis through differences in local tissue context and systemic signaling [27,28]. In the mesenteric lymph nodes, several innate immune-associated Hallmark features were represented among the DIABLO-derived signatures, consistent with responses within an immune compartment that remains functionally active in the athymic host. These findings emphasize the importance of anatomical context when selecting experimental models for liver tumors and suggest that ectopic models may capture different host–microbiome relationships than orthotopic models [24,25,27].

Previous preclinical studies have demonstrated links between the gut or intrahepatic microbiome and liver tumor development, immune responses, and metabolic alterations, predominantly using orthotopic liver tumor models [63,64]. Importantly, the anatomical context of tumor growth can substantially influence tumor phenotype and host responses, with orthotopic and ectopic models exhibiting distinct molecular and microenvironmental characteristics [65,66]. Thus, differences in tumor location may contribute to apparently divergent microbiome–tumor associations across preclinical studies. Our findings extend this concept by demonstrating that tumor anatomical location is associated with distinct host transcriptomic and microbial patterns, highlighting the importance of model context when interpreting microbiome–tumor interactions.

Our findings are broadly consistent with previous preclinical studies linking microbial alterations to liver tumor biology, including changes in inflammatory, metabolic, and tumor-associated pathways. However, the compartment-specific patterns observed here also highlight the importance of anatomical context. Orthotopic models provide a hepatic microenvironment with organ-specific stromal, vascular, and microbial interactions that differs from ectopic implantation, whereas ectopic models expose the tumor to a distinct tissue environment [67,68]. Previous comparisons of orthotopic and ectopic HCC xenografts have nevertheless reported variable effects of implantation site on tumor gene expression, suggesting that model- and tumor-specific factors may interact with anatomical context [65]. Thus, apparently discrepant microbiome–tumor associations across preclinical studies may partly reflect differences in tumor location and the resulting host–tumor microenvironment, and may help explain differences between reported observations.

Despite these insights, several limitations of our study should be acknowledged. First, while xenograft models enable a controlled investigation of complex tumor–host–microbiome interactions, they do not fully recapitulate the complexity of the human pediatric immune system or microbiome development [39,54,69,70]. This limitation is particularly relevant given the growing evidence that reciprocal interactions between the microbiome and adaptive immune system critically shape tumor progression and therapeutic responses across multiple cancer types [54].

Second, the host strain CAnN.Cg-Foxn1nu/Crl used is athymic and T-cell deficient [39]. The presence of interferon-associated signatures in the spleen and mesenteric lymph node should therefore be interpreted in the context of T-cell deficiency and cannot be assumed to represent adaptive antitumor immune responses [39,54]. Innate immune, myeloid, and stromal responses remain present in this model and may contribute to the observed transcriptional signatures. While this limits conclusions regarding adaptive immunity, it enables the investigation of tumor-associated host responses in the absence of mature T-cell-mediated activity [24,39].

Third, the inter-domain microbial associations identified by the multi-block framework represent statistical associations rather than causal relationships [52,53]. Although the cumulative ROC analyses showed high discriminatory performance for several feature blocks, these apparent classification estimates require cautious interpretation given the complexity of the integrated dataset and the limited cohort size relative to the dimensionality of the multi-omics dataset. Repeated cross-validation was used to assess model performance and reduce concerns about overfitting of the DIABLO model. Mechanistic studies involving targeted microbiome perturbation, metabolite profiling, microbiota transplantation, or gnotobiotic models will be required to determine whether the observed associations reflect direct functional interactions [28,29,54].

Fourth, although spatial profiling across ileum, colon, and feces provided high-resolution compartmental data, it represents a fixed snapshot that does not capture temporal dynamics during early oncogenesis [32,33]. Moreover, the critical influence of the host developmental stage, particularly during infancy and early childhood when human HB typically arises, cannot be fully recapitulated in adult murine systems [1,4,8,12].

Future studies should integrate longitudinal multi-omics, functional microbiome perturbation, and validation in pediatric patient cohorts to confirm causative links between gut ecosystems and HB biology [52,53,70]. Targeted manipulation of specific microbial and archaeal metabolic pathways (e.g., methanogenesis or ammonia oxidation) [41,42], combined with portal metabolomics [27,28], may help resolve the directionality of trans-portal interactions. Immune-competent, pediatric-relevant syngeneic models will be essential to address the adaptive immunological dimension of the gut–liver–tumor axis [29,39,54].

## 5. Conclusions

Overall, our findings support a model in which hepatoblastoma is associated with associated changes across tumor-intrinsic developmental programs, host transcriptional responses, and spatially structured microbial communities along the gut–liver axis [3,8,12,71]. The associations identified between microbial communities and host transcriptomic signatures are correlative and do not establish causality. Functional studies, including microbiota transplantation and targeted or untargeted metabolite profiling, will be required to determine whether the identified microbial features contribute directly to the observed host responses. Such mechanistic investigations represent important directions for future studies but were beyond the scope of the present work. The distinct patterns observed between orthotopic and ectopic models further suggest that anatomical context is an important factor of the host and microbial signatures captured by integrated multi-omics analysis. These findings provide a framework for investigating how tumor location, host responses, and microbial communities are associated in hepatoblastoma, while functional studies will be needed to determine the direction and biological consequences of these associations [23,25,29,54,69].

## Figures and Tables

**Table 1:** Tumor growth data and tissue weights of the control group (*n* = 6), the orthotopic group (*n* = 5) and the ectopic group (*n* = 6).

	Control		Orthotopic		Ectopic	
	Mean	Std. Deviation	Mean	Std. Deviation	Mean	Std. Deviation
Days until euthanasia	75.83	±27.67	94.2	±23.55	64.67	±18.08
Body weight (BW) [g]	25.23	±2.709	26.68	±1.731	24.73	±1.734
BW without Tumor [g]	25.23	±2.709	25.37	±2.316	23.18	±1.755
HB [mg]			1315	±1002	1550	±158.3
HB [% of BW]			5.46	±4.596	6.724	±0.9247
Lung [mg]	167.3	±33.6	134.5	±21.58	148.5	±27.17
Liver [mg]	1419	±252.4	1345	±77.95	1248	±166.4
Spleen [mg]	118.8	±39.82	90.94	±18.66	90.65	±18.56
Kidney [mg]	491.4	±93.63	470.1	±109.7	451.5	±38.75
Lymph node [mg]	8.72	±4.025	8.025	±5.001	6.533	±2.477
Triceps surae [mg]	130.9	±17.37	122.1	±31.42	117.7	±11.08
WAT gon [mg]	104.5	±25.03	78.92	±43.84	61.37	±29.01
WAT ing [mg]	77.87	±30.58	44.68	±33.07	5.867 *	±9.975
WAT ren [mg]	26.52	±9.135	37.16	±31.6	19.65	±9.889
WAT total [mg]	208.9	±40.88	160.8	±83.07	86.88 **^#^**	±31.21

HB: Hepatoblastoma; WAT: white adipose tissue; * *p* = 0.002; ^#^ *p* = 0.011.

**Table 2:** DIABLO cross-validation performance. Overall classification error, balanced error rate (BER), and class-specific error rates obtained from repeated 5-fold cross-validation with 100 repeats are shown for the two-component DIABLO model.

**Component**	**Overall Error**	**Overall BER**
Component 1	0.255	0.327
Component 2	0.098	0.135
**Component 2**	**Error**	
Ctrl	0	
Ectopic	0.026	
Orthotopic	0.38	

**Table 3:** Classification performance of the DIABLO components assessed by ROC analysis. AUC values and corresponding *p*-values are shown for each experimental group versus the remaining groups. Component 2 showed higher AUC values than Component 1 across all three comparisons.

	Comp1. AUC	Comp1. *p* Value	Comp2. AUC	Comp2. *p* Value
control vs. others	0.7105	0.362	0.9207	0.076
ectopic vs. Others	0.8077	0.217	0.9370	0.037
orthotopic vs. others	0.8490	0.210	0.9650	0.055

## Data Availability

Upon acceptance of the manuscript, all microbiome sequences and transcriptomics data will be made publicly available in the NCBI Gene Expression Omnibus (GEO) and Sequence Read Archive (SRA) repositories. Accession numbers will be provided prior to final publication.

## Supplementary Materials

The following supporting information can be downloaded at https://www.mdpi.com/article/10.3390/cancers18172854/s1, Figure S1: Global transcriptomic hallmark landscapes across all evaluated compartments. (A) Hierarchical clustered heatmap revealing global ssGSEA hallmark enrichment patterns across all five host murine organ blocks (*Mouse_Spleen*, *Mouse_Lymphnode*, *Mouse_Liver*, *Mouse_Lung*, *Mouse_Kidney*). (B) Unsupervised hierarchical clustering of the global hallmark expression profiles exclusive to the human xenograft tumor tissue, illustrating multi-directional transcriptomic scattering across replicate samples; Figure S2: Host renal transcriptomic stability under varying tumor topographies. (A) Boxplots illustrating the enrichment scores of the top 5 variable single-sample Gene Set Enrichment Analysis (ssGSEA) hallmarks within the host murine kidney (*Mouse_Kidney*). Individual data points represent replicate athymic CAnN.Cg-Foxn1nu/Crl mice across experimental groups: healthy controls (*Control*, green), ectopic subcutaneous tumors (*ectopic*, red), and orthotopic liver tumors (*orthotopic*, blue). Highly overlapping scores across cohorts demonstrate baseline transcriptomic stability. (B) Sparsity-aware hallmark co-regulatory interaction network within the renal parenchyma. Nodes represent specific hallmark pathways, sized by relative abundance and color-coded by the group exhibiting maximum enrichment (Control = green, ectopic = red, orthotopic = blue). Edges illustrate statistically significant co-regulatory correlations (*p* < 0.05). Pulmonary transcriptomic response maps system-wide cellular stress in the absence of mature T-lymphocytes. (C) Boxplots depicting relative ssGSEA enrichment scores for the top 5 variable hallmarks within the host murine lung (*Mouse_Lung*), showing a model-specific suppression of core cell-cycle machinery unique to ectopic subcutaneous tumor-bearing mice. (D) Sparsity-aware hallmark co-regulatory interactome of the lung parenchyma. The network topology unmasks a complete structural segregation of the mitotic block (comprising HM_E2F_TARGETS, HM_G2M_CHECKPOINT, and HM_MITOTIC_SPINDLE; blue, center-left) from the central host metabolic core (green), while systemic tumor-induced inflammatory cascades assemble into a highly integrated pathological module (red/blue, top); Table S1: Beta-diversity statistical validation matrix of mucosal and fecal biomes; Table S2: Linear Discriminant Analysis Effect Size (LEfSe) biomarker profiles; Table S3: single-sample Gene Set Enrichment Analysis (ssGSEA) results for every single hallmark; Table S4: DIABLO multi-block cross-omics feature loading weights; Table S5: RNA integrity of samples included in the transcriptomic analysis; Table S6: Numbers of biological replicates included in the transcriptomic and microbiome analyses. File S1: ARRIVE Author Checklist.
