# Supplementary material for: Tumor Topography Remodels TME Signalling via Compartment-Specific Host–Microbiome–Tumor Crosstalk in Hepatoblastoma
Source: Cancers (Basel). 2026 Sep 3;18(17):2854. doi: 10.3390/cancers18172854 (PMC13565734; doi:10.3390/cancers18172854)
Supplement: Supplementary file 1 [file cancers-18-02854-s001.zip › Supplementary File S1-ARRIVE Author Checklist - Full.pdf]

NOTE: Please save this file locally before filling in the table, DO NOT work on the file within your internet browser as changes will not be saved. Adobe Acrobat Reader (available free here) is recommended for completion.

The ARRIVE guidelines 2.0: author checklist

The ARRIVE Essential 10

These items are the basic minimum to include in a manuscript. Without this information, readers and reviewers cannot assess the reliability of the findings.

Item		Recommendation	Section/line number, or reason for not reporting
**Study design**	1	For each experiment, provide brief details of study design including:<br>a. The groups being compared, including control groups. If no control group has been used, the rationale should be stated.<br>b. The experimental unit (e.g. a single animal, litter, or cage of animals).	lines 201-206<br>one single housed animal is one unit
**Sample size**	2	a. Specify the exact number of experimental units allocated to each group, and the total number in each experiment. Also indicate the total number of animals used.<br>b. Explain how the sample size was decided. Provide details of any *a priori* sample size calculation, if done.	3 groups with 6 animals each<br>Sample sizes were based on previous experience with this experimental model. The number of control animals was minimized in accordance with the 3Rs principle while maintaining statistical comparability.
**Inclusion and exclusion criteria**	3	a. Describe any criteria used for including and excluding animals (or experimental units) during the experiment, and data points during the analysis. Specify if these criteria were established *a priori*. If no criteria were set, state this explicitly.<br>b. For each experimental group, report any animals, experimental units or data points not included in the analysis and explain why. If there were no exclusions, state so.<br>c. For each analysis, report the exact value of *n* in each experimental group.	No predefined inclusion or exclusion criteria were established prior to the study<br>one human orthotopic tumor sample did not amplyfy due to poor extraction and was therefore excluded.<br>Control n=6, Ectopic n=6, Orthotopic n=5 except for human tumor samples where n=4
**Randomisation**	4	a. State whether randomisation was used to allocate experimental units to control and treatment groups. If done, provide the method used to generate the randomisation sequence.<br>b. Describe the strategy used to minimise potential confounders such as the order of treatments and measurements, or animal/cage location. If confounders were not controlled, state this explicitly.	Animals were allocated to the study groups to achieve a comparable body weight distribution between groups.<br>positioned according to ascending animal identification numbers, resulting in an interspersed distribution of animals from different study groups within the animal facility. No additional measures to control potential confounding factors were implemented.
**Blinding**	5	Describe who was aware of the group allocation at the different stages of the experiment (during the allocation, the conduct of the experiment, the outcome assessment, and the data analysis).	The principal investigator was aware of the group allocation during animal allocation, conduct of the experiment, outcome assessment, and data analysis. No blinding was performed.
**Outcome measures**	6	a. Clearly define all outcome measures assessed (e.g. cell death, molecular markers, or behavioural changes).<br>b. For hypothesis-testing studies, specify the primary outcome measure, i.e. the outcome measure that was used to determine the sample size.	lines 259-262, 280-294,<br>Not applicable. This was an exploratory study and no primary outcome measure was defined for sample size determination.
**Statistical methods**	7	a. Provide details of the statistical methods used for each analysis, including software used.<br>b. Describe any methods used to assess whether the data met the assumptions of the statistical approach, and what was done if the assumptions were not met.	lines 224-320,<br>Because of the smal sample size not normally distributed data were assumed.
**Experimental animals**	8	a. Provide species-appropriate details of the animals used, including species, strain and substrain, sex, age or developmental stage, and, if relevant, weight.<br>b. Provide further relevant information on the provenance of animals, health/immune status, genetic modification status, genotype, and any previous procedures.	line 175-184<br>line 201-207
**Experimental procedures**	9	For each experimental group, including controls, describe the procedures in enough detail to allow others to replicate them, including:<br>a. What was done, how it was done and what was used.<br>b. When and how often.<br>c. Where (including detail of any acclimatisation periods).<br>d. Why (provide rationale for procedures).	line 201-209;<br>line 209-217<br>The concentration and volume of the inoculum were determined in a preliminary pilot study and explicitly approved by the competent authority to ensure reliable tumor [illegible]
**Results**	10	For each experiment conducted, including independent replications, report:<br>a. Summary/descriptive statistics for each experimental group, with a measure of variability where applicable (e.g. mean and SD, or median and range).<br>b. If applicable, the effect size with a confidence interval.	See table 1<br>not applicable

The Recommended Set

These items complement the Essential 10 and add important context to the study. Reporting the items in both sets represents best practice.

Item		Recommendation	Section/line number, or reason for not reporting
**Abstract**	11	Provide an accurate summary of the research objectives, animal species, strain and sex, key methods, principal findings, and study conclusions.	line 31-54
**Background**	12	a. Include sufficient scientific background to understand the rationale and context for the study, and explain the experimental approach.	line 31-35, 169-172
		b. Explain how the animal species and model used address the scientific objectives and, where appropriate, the relevance to human biology.	line 164-168
**Objectives**	13	Clearly describe the research question, research objectives and, where appropriate, specific hypotheses being tested.	Line 169-172
**Ethical statement**	14	Provide the name of the ethical review committee or equivalent that has approved the use of animals in this study, and any relevant licence or protocol numbers (if applicable). If ethical approval was not sought or granted, provide a justification.	Austrian veterinary board (2024-0.307.647 and 2025-0.281.987)
**Housing and husbandry**	15	Provide details of housing and husbandry conditions, including any environmental enrichment.	line178-184; mice had nestlets, wodden gnawir
**Animal care and monitoring**	16	a. Describe any interventions or steps taken in the experimental protocols to reduce pain, suffering and distress.	line212-215 tissue sampling and anesthesia were conducted in two separate rooms to aviod distress.
		b. Report any expected or unexpected adverse events.	line 322-323
		c. Describe the humane endpoints established for the study, the signs that were monitored and the frequency of monitoring. If the study did not have humane endpoints, state this.	line 182-184; endpoints were defined in the score sheet and approved by the Austrian veterinary board
**Interpretation/ scientific implications**	17	a. Interpret the results, taking into account the study objectives and hypotheses, current theory and other relevant studies in the literature.	Line 706-809; 835-849
		b. Comment on the study limitations including potential sources of bias, limitations of the animal model, and imprecision associated with the results.	line 811-834
**Generalisability/ translation**	18	Comment on whether, and how, the findings of this study are likely to generalise to other species or experimental conditions, including any relevance to human biology (where appropriate).	line 846-849
**Protocol registration**	19	Provide a statement indicating whether a protocol (including the research question, key design features, and analysis plan) was prepared before the study, and if and where this protocol was registered.	A study plan including the research question, experimental design, analysis plan, and a time schedule was prepared prior to the start of the study. The plan was not formally registered in a public registry, but was documented and implemented within the animal facility to ensure adherence to the planned study timeline and procedures.
**Data access**	20	Provide a statement describing if and where study data are available.	Upon acceptance of the manuscript, all microbi
**Declaration of interests**	21	a. Declare any potential conflicts of interest, including financial and non-financial. If none exist, this should be stated.	The authors declare no competing financial or personal interests.
		b. List all funding sources (including grant identifier) and the role of the funder(s) in the design, analysis and reporting of the study.	This study was funded by a grant from the Medical University of Graz and MEFOgraz (" Startförderung ” , project number: 8151) to BO.The funders had no role in study design, data collection and analysis, decision to publish, or preparation of the manuscript
